# Supplementary material for: Differential analysis of RNA structure probing experiments at nucleotide resolution: uncovering regulatory functions of RNA structure
Source: Nat Commun. 2022 Jul 22;13:4227. doi: 10.1038/s41467-022-31875-3 (PMC9307511; doi:10.1038/s41467-022-31875-3)
Supplement: Supplementary file 1 — Supplementary Information [file 41467_2022_31875_MOESM1_ESM.pdf]

Differential Analysis of RNA Structure Probing Experiments at Nucleotide Resolution: Uncovering Regulatory Functions of RNA Structure

Contents

Supplementary Note I 5
Normalization module: Theoretical justifications 5
Supplementary Note II 8
Normalization module: Empirical validations 8
Supplementary Note III 10
Normalization module: Sensitivity analysis 10
Supplementary Note IV 11
Normalization module: Performance comparison with existing normalization methods 11
Supplementary Note V 13
Scan module: Sensitivity analysis of the Q function in scan statistic 13
Supplementary Note VI 14
Illustration of the definition of average nucleotide distance 14
Supplementary Note VII 15
Prediction results for the Flu and RRE dataset at varying cutoffs 15
Supplementary Methods 16
Normalization module: Determination of the structurally invariant set *S* 16
Scan module: Monte Carlo approach controlling for family-wise error rate 17
Simulations: Three types of reactivity models used to simulate reactivities 18
Supplementary Tables 19
Supplementary Table 1 19
Supplementary Table 2 19
Supplementary Table 3 19
Supplementary Table 4 20
Supplementary Table 5 20
Supplementary Table 6 21
Supplementary Table 7 22
Supplementary Table 8 22

Supplementary Figures 23
Supplementary Figure 1 23
Supplementary Figure 2 24
Supplementary Figure 3 25
Supplementary Figure 4 26
Supplementary Figure 5 27
Supplementary Figure 6 28
Supplementary Figure 7 29
Supplementary Figure 8 30
Supplementary Figure 9 31
Supplementary Figure 10 32
Supplementary Figure 11 33
Supplementary Figure 12 34
Supplementary Figure 13 35
Supplementary Figure 14 36
Supplementary Figure 15 37
Supplementary Figure 16 38
Supplementary Figure 17 39
Supplementary Figure 18 40
Supplementary Figure 19 41
Supplementary Figure 20 42
Supplementary Figure 21 43
Supplementary Figure 22 44
Supplementary Figure 23 45
Supplementary Figure 24 46
Supplementary Figure 25 47
Supplementary Figure 26 48
Supplementary Figure 27 49
Supplementary Figure 28 50

Supplementary Figure 29 51

Supplementary Figure 30 52

Supplementary Figure 31 53

Supplementary Figure 32 54

Supplementary Figure 33 55

Supplementary Figure 34 56

References.. 57

Supplementary Note I

Normalization module: Theoretical justifications

For nucleotide position *j*, consider a generative model concerning its secondary structure and the observed reactivity value r_j at it. r_j is composed of the underlying reactive level $r_j^* = \mathrm{E}r_j$, which is a constant, and an error term ϵ_j capturing the deviation from the observed level and the expected level, *i.e.*,

$$r_j = r_j^* + \epsilon_j, \mathrm{E}\epsilon_j = 0.$$

Assume the underlying reactive level r_j^* is determined by the secondary structure of nucleotide position *j*, *i.e.*,

$$r_j^* = f(p_j),$$

in which $p_j \in [0,1]$ is the proportion of unpaired nucleotides at position j in the co-existing structure conformations, and f is an unknown function.

Therefore, given two experiments A and B, we have

$$r_j^A = f^A(p_j^A) + \epsilon_j^A,$$

and

$$r_j^B = f^B(p_j^B) + \epsilon_j^B.$$

In differential analysis, we want to compare p_j^A and p_j^B by comparing the observed values r_j^A and r_j^B. The obstacles are the heterogeneity in experiments, *i.e.*, f^A and f^B are not necessarily identical.

In practice, substantial difference in f^A and f^B is observed in SP data, as in many other high-throughput experiments. For example, Supplementary Figure 1 shows the MA plot for within-condition replicates of the negative control dataset Control 5. MA plot is frequently used in normalization literature to visualize systematic bias[1-4]. For within-condition replicates (A1 and A2), there is no biological difference in RNA secondary structure, and thus $p_j^{A1} = p_j^{A2}$. Therefore, if $f^{A1} = f^{A2}$, we have $r_j^{A1} \approx r_j^{A2}$, and thus the M value $\log \frac{r_j^{A1}}{r_j^{A2}} \approx 0$ regardless of the Average value $\frac{\log r_j^{A1} + \log r_j^{A2}}{2}$. However, significant dependency of M values on A values is observed (p value = 6.6e-4 in linear regression $M \sim A$), suggesting the difference between f^{A1} and f^{A2}.

Similar patterns of MA plot are frequently reported in related analysis of high throughput sequencing data, including ChIP-Seq[2] and ATAC-Seq[5]. Normalization methods for ChIP-Seq and ATAC-Seq data have been developed to correct differences in sequencing depth and signal to noise ratio between replicates, including MAnorm[2] and S3norm[5]. Inspired from these methods, we assume non-linear functional forms for f^A and f^B (Equation 1 and 2), introducing scaling factors α^A and α^B to characterize the sequencing depths of experiments A and B, and power factors β^A and β^B to characterize signal to noise ratios of experiments A and B.

$$f^A(p_j^A) = \alpha^A (p_j^A)^{\beta^A}, \qquad \text{(Equation 1)}$$

$$f^B(p_j^B) = \alpha^B (p_j^B)^{\beta^B}. \qquad \text{(Equation 2)}$$

Here, signal to noise ratio refers to the magnitude of difference between the underlying reactive levels at nucleotides with different secondary structures, *i.e.*, how the experiment distinguishes p_j's levels between 0 and 1. Difference in signal to noise ratios of experiments can be introduced by difference in many biological or technical factors[6, 7]. For example, the reaction efficiency of the SP reagent may be different when probed in different cellular context. Another example is that when transcripts in experiment A is more structured than experiment B, the SP regent may be allocated to less/more nucleotides in A compared to B, depending on the reaction preference of the SP reagent. An illustrative example is shown in Supplementary Figure 26. If we can correct the difference between f^A and f^B , we can normalize the observed reactivities and enable differential analysis.

To correct the difference between f^A and f^B, we can transform f^A into

$$\text{normalized } f^A = \alpha^B * \left(\left(\frac{f^A}{\alpha^A} \right)^{\frac{1}{\beta^A}} \right)^{\beta^B}, \qquad \text{(Equation 3)}$$

so that when $p_j^A = p_j^B$, we have

$$\text{normalized } f^A(p_j^A) = f^B(p_j^B),$$

thus $r_j^A \approx r_j^B$. To this end, we need to learn the transformation in Equation 3 from data, which can be simplified into

$$\text{normalized } f^A = \left(\alpha^B * \left(\frac{1}{\alpha^A} \right)^{\frac{\beta^B}{\beta^A}} \right) * (f^A)^{\frac{\beta^B}{\beta^A}} = \alpha * (f^A)^{\beta}, \qquad \text{(Equation 4)}$$

in which $\alpha = \alpha^B * \left(\frac{1}{\alpha^A}\right)^{\frac{\beta^B}{\beta^A}}$ and $\beta = \frac{\beta^B}{\beta^A}$. Applying log transformation to both sides of Equation 4, we have

$$\log(\text{normalized } f^A) = \log \alpha + \beta * \log(f^A).$$

Therefore, we only need to learn $\log \alpha$ and β from data to correct the difference between f^A and f^B. The above is the theoretical motivation of our transformation approach. We note that the robust regression step of the Normalization module is similar to the techniques used by MAnorm and S3norm to correct for differences in sequencing depth and signal to noise ratio between replicates in ChIP-Seq data and ATAC-Seq data.

Supplementary Note II

Normalization module: Empirical validations

We manipulated the sequencing depth and signal to noise ratio in synthetic and real reactivities to evaluate whether the normalization procedure removes unwanted variation and retains biological signal. Specifically, we showed the boxplot of M values[2] (Equation 5) to visualize the normalization effect. Note M value is frequently used to visualize and evaluate normalization performance in high-throughput experiments. We expect a successful normalization procedure to shift the distribution of M values to be centered around 0 at non-SVR nucleotide positions.

$$\text{M value at position } j \ = \ \log\frac{x_j}{y_j}, \qquad \text{(Equation 5)}$$

where x_j and y_j are reactivities at position *j* of between-condition replicates.

(I) Negative control datasets. We took reactivities of Control 1, constructed by contrasting the replicates of the condition without fluoride ions of the Flu dataset, and introduced difference in sequencing depth and signal to noise ratio between conditions by transforming reactivities of group B (denoted by r_B) into $\alpha * r_B^{\beta}$. When the raw reactivities were disturbed at different levels of α and β, the Normalization module consistently shifted the median of M values towards 0 (Supplementary Figure 27).

(II) Positive control datasets. We constructed the dataset by adding real differential signals into the synthetic negative control dataset Control 1. To elaborate, group A and group B reactivities in Control 1 are biological replicates of the condition without fluoride ions of the Flu dataset. We replaced group B reactivities of Control 1 in the annotated SVRs of the Flu dataset with the reactivities of the condition with fluoride ions at the same nucleotide positions. Thus, in the synthetic positive control datasets, the reactivities in the annotated SVRs represent biological differential signals, while the reactivities in the other nucleotide positions do not manifest structure difference. Similarly, as in (I), we introduced difference in sequencing depth and signal to noise ratio between the contrasted groups by transforming reactivities of group B (denoted by r_B) into $\alpha * r_B^{\beta}$. DiffScan consistently shifted the median of M values at non-SVR nucleotide positions towards 0, while the distribution of M values in true SVRs can be distinguished from that of non-SVRs (Supplementary Figure 28).

(III) Benchmark datasets. Next, we evaluated the normalization method in the Flu dataset, which has raw count data and annotated secondary structure[8] in each of the two compared conditions. We introduced difference in sequencing depth by amplifying the reverse transcription (RT) counts of condition B at position j (denoted by $\mathrm{RT_j}$) into $f_j * \mathrm{RT_j}$, in which f_j is sampled from $\mathrm{N}(2,0.5^2)$ to reflect random fluctuations across nucleotides. Raw reactivities are severely affected by the imbalanced depth of sequencing in the two conditions, and DiffScan

successfully shifted the median of M values at non-SVR nucleotide positions towards 0 and retained the differential signal in the annotated SVRs (Supplementary Figure 29a). Then, we introduced difference in signal to noise ratio (*i.e.*, the contrast between the reactive levels of nucleotides with and without structure modifications in one condition) by amplifying the RT counts of condition B with f_j at the unpaired nucleotide positions. Similarly, DiffScan shifted the median of M values at non-SVR nucleotide positions towards 0 and retained the differential signal in the annotated SVRs (Supplementary Figure 29b).

To conclude, in the presence of difference in sequencing depth and signal to noise ratio between the two compared conditions, our normalization procedure removes unwanted variation and retains the differential signal in SVRs.

Supplementary Note III

Normalization module: Sensitivity analysis

We conducted sensitivity analysis of the Normalization module for two scenarios. Scenario 1: we investigated the performance of Normalization when the pivot set is mis-specified by including nucleotides with structural variations. Scenario 2: we perturbed the reactivities by introducing varying levels of sequencing depth and signal to noise ratio along the transcript.

For scenario 1, we gradually mixed 0%, 10%, 20%, 30%, 40%, and 50% of the nucleotide positions in annotated SVRs in the Flu dataset into the perfect pivot set *S* (the annotated non-SVR nucleotide positions) and performed normalization. The normalized reactivities highly resembles each other (Supplementary Figure 30a)**,** with an average correlation of 0.998. We further evaluated the performance of normalization with the M values[2] (Equation 5). When the pivot set is contaminated at different levels, the Normalization module consistently shifts the median of M values towards zero (Supplementary Figure 30b). Thus, we conclude the performance of the Normalization module is robust to the misspecification of the structurally invariant set.

For scenario 2, we transformed reactivities in Control 1 (two replicates, say A and B, randomly selected from the Flu dataset in the absence of fluoride) to introduce local variations of sequencing depth and signal to noise ratio. In detail, we partitioned the transcript into four segments of equal length (25 nt), and for segment *i*, we sampled parameters α_i and β_i, from the empirical distributions obtained from the Flu dataset (Supplementary Figure 31a-b, see Note below). The reactivities of segment i in group B (denoted by $r_{B;i}$) were transformed into $\alpha_i * r_{B;i}^{\beta_i}, 1 \leq i \leq 4$. By setting different α's and β's for each segment, the sequencing depth and signal to noise ratio are made different from segment to segment. Next, we applied the Normalization module to fit global transformation factors $\hat{\alpha}, \hat{\beta}$ for the transcript, and also implemented an oracle normalization that the transformation factors were fitted per segment. The random perturbation and subsequent normalization procedure were repeated 100 times. Regarding to the M values, the Normalization module was robust and the performance was comparable to the oracle normalization results (Supplementary Figure 31c).

We conclude that the normalization approach we proposed is robust to model misspecifications such as contaminated pivot set and locally varying parameters of sequencing depth and signal to noise ratios.

Note: We fitted the empirical distributions of α and β from different segments of the Flu dataset. To elaborate, we slid through the transcript with 1-step moving widows of 30 nt wide. Within each window, we fitted α and β using the Normalization module.

Supplementary Note IV

Normalization module: Performance comparison with existing normalization methods

We compared the Normalization module of DiffScan with the 2%-8% normalization method[9] and the BUM-HMM method[10] in the negative control datasets and the benchmark datasets (see Supplementary Methods).

Comparing with BUM-HMM:

The empirical P value calculated by BUM-HMM can be considered as a normalization step, as it processes raw read counts and outputs scaled values (between 0-1). However, BUM-HMM was developed and optimized to sensitively identify nucleotide modification in a specific condition. When used as a normalization step in differential analysis, BUM-HMM independently processes read counts from two conditions. In contrast, the Normalization module in DiffScan combines reactivities from two conditions and normalize them relative to one another, towards the goal of removing non-biological variability between two conditions. Another advantage of DiffScan is that it works reasonably well when there are no within-condition replicates, while replicates are indispensable to the application of BUM-HMM.

To quantify the normalization effect of different methods, we use the evaluation metric of M values[2] (Equation 5), which are commonly used for evaluating normalization analysis. Normalization analysis is expected to shift the center of the M values in non-SVR nucleotide positions around zero to remove systematic bias. In the negative control datasets, the Normalization module consistently shifts the median of M values towards zero (Supplementary Figure 32a). The distribution of M values of the normalized reactivities by BUM-HMM are far more extensive (Supplementary Figure 32b). The posterior probabilities of modification have two modes, centered around 0 and 1 (Supplementary Figure 32c), which cannot be easily combined with downstream statistical tests based on normal distributions. We would like to clarify that the above comparisons and discussions is not a critique of BUM-HMM, as the scope of BUM-HMM is to characterize the posterior probability of modification in single condition. Its output for two conditions separately is not immediately comparable in differential analysis, which has been discussed in Marangio *et al*[10, 11]. Note that BUM-HMM is not applicable to Control 3-6 due to lack of within-condition replicates of raw count data. In the benchmark Flu dataset, DiffScan shifts the median of M values at non-SVR nucleotide positions towards zero, and meanwhile retains the differential signal in SVR nucleotide positions (Supplementary Figure 33a, c). The M values of the normalized reactivities by BUM-HMM are dispersed at both non-SVR and SVR nucleotide positions (Supplementary Figure 33b). BUM-HMM is not applicable to the benchmark RRE dataset due to lack of within-condition-replicates of raw count data.

Comparing with 2%-8% normalization:

Similarly, we evaluated the 2%-8% normalization via the distribution of normalized reactivities. In the datasets Control 1-6, its performance is comparable to DiffScan in Control 1, 3, 4, and 6, and a significant deviation of its M values from zero can be observed for Control 2 and 5 (Supplementary Figure 32a). In the benchmark datasets, the M values of its normalized reactivities at non-SVR nucleotide positions show a deviation from zero (Supplementary Figure 33a, c). In contrast, the Normalization module of DiffScan works consistently well in all tests.

Supplementary Note V

Scan module: Sensitivity analysis of the Q function in scan statistic

There are two tuning parameters in DiffScan: the radius of local windows (denoted by *r*) for smoothed calculation of positional differential signal, and the parameter associated with the penalty term (denoted by γ, *i.e.*, $Q(R) = \frac{-\sum_{j \in R} \log(p_j)}{|R|^{\gamma}}$) in the scan statistic. In the analysis of the simulated datasets, we set *r* to 2 nt and γ to 0.5 as their default values. We also conducted a sensitivity analysis wherein we tested DiffScan with the simulated datasets using eight other settings of (r, γ). In each setting, we calculated the mean precision of the predicted SVRs at recall values below 0.05. The coefficient of variation for the mean precisions corresponding to different tuning parameters is below 0.15 for all of the examined scenarios in the simulations (Supplementary Table 8), showing the robustness of DiffScan regarding to the tuning parameters. Notably, DiffScan consistently outperformed dStruct and deltaSHAPE in these tested parameter settings (Supplementary Fig. 34).

Supplementary Note VI

Illustration of the definition of average nucleotide distance

For each nucleotide position in a predicted SVR, the distance to true SVRs is the number of nucleotides between itself and the nearest nucleotide in all true SVRs. The nucleotide distance of a predicted SVR is calculated by taking the average of the distances of all its nucleotides. As illustrated in Supplementary Figure 9, the true SVR covers nucleotide positions 6 nt – 8 nt in the transcript, and Detection result 1 reports a region covering nucleotide position 5 nt – 9 nt. Then the nucleotide distances for Detection result 1 are 1, 0, 0, 0, 1, and the average distance is $\frac{1+0+0+0+1}{5} = 0.4$. In the same way, the average distance for Detection result 2 is $\frac{5+0+0+0+5}{5} = 2$. By definition, the nucleotide distance rewards accurate boundary mapping of predicted SVRs to true SVRs, since predicted SVRs within true SVRs are given a minimum distance 0. The nucleotide distance assigns a larger penalty to the predicted SVRs that are far away from any true SVRs, while in the calculation of specificity or Jaccard index, off-target predictions are not distinguished at all. The advantage of DiffScan is its accurate boundary mapping of SVRs, thus we consider the nucleotide distance an informative evaluation metric. For example, in Supplementary Figure 9, Detection result 1 and 2 both reported two false nucleotides. However, the false discoveries in Detection result 2 are biologically more misleading. As expected, Detection result 2 has higher nucleotide distances (2) than Detection result 1 (0.2).

Supplementary Note VII

Prediction results for the Flu and RRE dataset at varying cutoffs

For the Flu dataset, we have reported the results of the top-20 ranked nucleotides by different methods in the main text. 49% of the top-40 nucleotides identified by DiffScan are in annotated SVRs. 40% of the top-40 nucleotides identified by diffBUM-HMM are in annotated SVRs. For dStruct, 49% of the top-40 nucleotides overlap with annotated SVRs.

For the RRE dataset, 86% of the top-20 and 87% of the top-40 nucleotides identified by DiffScan are in the annotated SVRs. deltaSHAPE identified four regions, of 15 nucleotides in sum, and 47% of the nucleotides overlap with annotated SVRs. For dStruct, 78% of the top-20 and 58% of the top-40 nucleotides are in the annotated SVRs. diffBUM-HMM, PARCEL, and RASA were not applicable to the RRE dataset.

Supplementary Methods

Normalization module: Determination of the structurally invariant set *S*

S is determined as the nucleotide positions with reactivities uniformly high or uniformly low across all replicates. These positions are probably simultaneously paired or simultaneously unpaired in both conditions. Technically, given two parameters $(l, u), 0 \leq l \leq 0.5 \leq u \leq 1$,

$$S_i^{\text{A,high}} = \left\{ j | r_{ij}^A \geq \text{quantile}\left(\left\{r_{it}^A, 1 \leq t \leq n\right\};\ u\right)\right\}$$

consists of nucleotide positions with high reactivities in replicate i from condition A, and

$$S_k^{\text{B,high}} = \left\{ j | r_{kj}^B \geq \text{quantile}\left(\left\{r_{kt}^B, 1 \leq t \leq n\right\};\ u\right)\right\}$$

consists of nucleotide positions with high reactivities in replicate k from condition B. Therefore,

$$S^{\text{high}} = \left(\cap_{i=1}^{n_A} S_i^{\text{A,high}}\right) \cap \left(\cap_{k=1}^{n_B} S_k^{\text{B,high}}\right)$$

consists of nucleotide positions with uniformly high reactivities across all replicates. Similarly,

$$S^{\text{low}} = \left(\cap_{i=1}^{n_A} S_i^{\text{A,low}}\right) \cap \left(\cap_{k=1}^{n_B} S_k^{\text{B,low}}\right)$$

consists of nucleotide positions with uniformly low reactivities across all replicates, where

$$S_i^{\text{A,low}} = \left\{ j | r_{ij}^A \leq \text{quantile}\left(\left\{r_{it}^A, 1 \leq t \leq n\right\};\ l\right)\right\}$$

and

$$S_k^{\text{B,low}} = \left\{ j | r_{kj}^B \leq \text{quantile}\left(\left\{r_{kt}^B, 1 \leq t \leq n\right\};\ l\right)\right\}.$$

Then the invariant set is determined as

$$S = S^{\text{high}} \cup S^{\text{low}}.$$

At last, (l, u) is selected by solving the following optimization problem with grid searching.

$$(l, u) = \underset{\substack{0.05 \leq l \leq 0.4 \\ 0.6 \leq u \leq 0.95 \\ |S| \geq \max\left(10, \frac{n}{20}\right)}}{\text{argmax}} \quad \text{spearman correlation}\left(\overline{r_j^A}, \overline{r_j^B}\right)_{j \in S}.$$

The idea is that when *nucleotide* positions with structural variations are added to *S*, the spearman correlation of between-group reactivities in *S* should decrease.

Scan module: Monte Carlo approach controlling for family-wise error rate

At significance level α, we first calculate a threshold h_α for the scan statistic $Q(R)$ from Monte Carlo sampling. Then we implement the following algorithm which outputs the predicted SVRs by DiffScan.

Input: $\mathcal{R} = \{L_{\min} \leq |R| \leq L_{\max}\},\ \{Q(R') \mid R' \in \mathcal{R}\}$.
1: $\mathrm{SVR} \leftarrow \emptyset$.
2: **while** $\mathcal{R} \neq \emptyset$ **do**
3: $\quad R \leftarrow \underset{R' \in \mathcal{R}}{\operatorname{argmax}}\, Q(R')$.
4: $\quad$ **if** $Q(R) < h_\alpha$ **then**
5: $\qquad$ **return** SVR
6: $\quad$ **else**
7: $\qquad \mathrm{SVR} \leftarrow \mathrm{SVR} \cup \{R\}$.
8: $\qquad \mathcal{R} \leftarrow \{R' \in \mathcal{R} \mid R' \text{ does not overlap with } R\}$.
9: $\quad$ **end if**
10: **end while**
11: **return** SVR

To calculate the threshold h_α, we sample $\widetilde{p_J} \sim \mathrm{i.i.d.\,U}(0,1), 1 \leq j \leq n$ and compute the corresponding scan statistic values

$$\widetilde{Q(R)} = \frac{-\Sigma_{j\in R}\log\left(\widetilde{p_J}\right)}{\sqrt{|R|}}, R \in \mathcal{R}$$

and the extreme statistic

$$\widetilde{Q_{\max}} = \max\left\{\widetilde{Q(R)} \mid R \in \mathcal{R}\right\}.$$

This process is repeated N times to get N replications of $\widetilde{Q_{\max}}$, from which we calculate

$$h_\alpha = \mathrm{quantile}\left\{\widetilde{Q_{\max}}; 1-\alpha\right\}.$$

In practice, we cut the transcript and/or the transcriptome into 100 nt segments and then enumerate contiguous regions with a minimum length $L_{\min} = 1$ nt and a maximum length $L_{\max} = 20$ nt in the 100 nt segments. Note that $L_{\max} = 20$ nt is not the upper bound of length of finally predicted SVRs, since DiffScan would predict multiple SVRs which might adjoin each other within long SVRs.

Simulations: Three types of reactivity models used to simulate reactivities

Two types of distributions of SHAPE reactivities were fitted in an existing literature[12] from two independent sources.

Cordero et al.[13]:

$$\text{reactivity}_{\text{paired}} \sim \text{Generalized Extreme Value (GEV) distribution}(\mu = 0.0947, \sigma = 0.0672, \epsilon = 0.2352),$$

$$\text{reactivity}_{\text{unpaired}} \sim \text{GEV}(\mu = 0.2198, \sigma = 0.1852, \epsilon = 0.5426).$$

Sükösd et al.[14]:

$$\text{reactivity}_{\text{paired}} \sim \text{GEV}(\mu = 0.0523, \sigma = 0.0680, \epsilon = 0.8681),$$

$$\text{reactivity}_{\text{unpaired}} \sim \text{exponential}(\lambda = 1.4638).$$

We fitted reactivity distributions for paired and unpaired nucleotides characterizing the statistical nature of reactivities acquired using the icSHAPE platform utilizing reactivities of 100 transcripts we selected from an icSHAPE dataset[15]:

$$\text{reactivity}_{\text{paired}} \sim \pi_1 \delta_0 + (1 - \pi_1) \exp\left(\text{Normal}(\mu_1, \sigma_1^2)\right),$$

$$\text{reactivity}_{\text{unpaired}} \sim \pi_2 \delta_0 + (1 - \pi_2) \exp\left(\text{Normal}(\mu_2, \sigma_2^2)\right),$$

in which δ_0 is a point mass at 0 and $\widehat{\pi_1} = 0.68, \widehat{\mu_1} = -3.40, \widehat{\sigma_1^2} = 3.53, \widehat{\pi_2} = 0, \widehat{\mu_2} = -2.69, \widehat{\sigma_2^2} = 0.97$. ($\mu_1, \sigma_1^2$ and μ_2, σ_2^2 were estimated using a two-component Gaussian finite mixture model[16] from the nonzero values of the icSHAPE reactivities. π_1 and π_2 were further estimated combined with the frequency of paired and unpaired nucleotides in the simulated secondary structures.)

Supplementary Tables

Supplementary Table 1 Median length of the top-1,000 predicted SVRs by dStruct with minimum search length of 5 nt in the simulated datasets. Rows: types of reactivity models. Columns: levels of strength of differential signal.

	Low	Medium	High
Cordero *et al.* 2012	185.5	537.5	620
icSHAPE	27	41	49
Sükösd *et al.* 2013	157.5	396.5	590.5

Supplementary Table 2 Median length of the top-1,000 predicted SVRs by dStruct with minimum search length of 1 nt in the simulated datasets. Rows: types of reactivity models. Columns: levels of strength of differential signal.

	Low	Medium	High
Cordero *et al.* 2012	66	128	233
icSHAPE	8	11	14
Sükösd *et al.* 2013	45	76	122.5

Supplementary Table 3 Median length of the top-1,000 predicted SVRs by dStruct with minimum search length of 11 nt in the simulated datasets. Rows: types of reactivity models. Columns: levels of strength of differential signal.

	Low	Medium	High
Cordero *et al.* 2012	177.5	573	616
icSHAPE	119	216	348.5
Sükösd *et al.* 2013	184.5	561.5	615.5

Supplementary Table 4 Applicability of different methods to the negative control datasets and benchmark datasets.

Dataset	DiffScan	deltaSHAPE	diffBUM-HMM	dStruct	PARCEL	RASA
Control 1	√	√	√	√	√	√
Control 2	√	√	√	√	√	√
Control 3	√	×(*)	×(&)	√	×(&)	×(&)
Control 4	√	×(*)	×(&)	√	×(&)	×(&)
Control 5	√	√	×(#)	×	×(#)	√
Control 6	√	√	×(#)	×	×(#)	√
Flu	√	√	√	√	√	√
RRE	√	√	×(&)	√	×(&)	×(&)

&: not applicable due to lack of count data;

#: not applicable due to lack of multiple within-condition replicates;

*: not applicable due to lack of count data and multiple within-condition replicates.

Supplementary Table 5 Number of predicted SVRs by the compared methods in the negative control datasets. DiffScan: family-wide error rate (FWER) < 0.05; diffBUM-HMM: posterior probability > 0.95; dStruct: false discovery rate (FDR) < 0.05. NA indicates that the method was not applicable for the dataset.

Dataset	DiffScan	deltaSHAPE	diffBUM-HMM	dStruct	PARCEL	RASA
Control 1	1	3	7	0	0	0
Control 2	0	2	4	0	0	1
Control 3	0	NA	NA	1	NA	NA
Control 4	0	NA	NA	0	NA	NA
Control 5	0	15	NA	NA	NA	0
Control 6	0	11	NA	NA	NA	0

Supplementary Table 6 RBP motifs enriched in the DiffScan-predicted SVRs from the Np versus Cy comparison (FDR < 0.05). P values were calculated using the one-sided Wilcoxon signed-rank test and adjusted with the Benjamini-Hochberg method.

Gene name	Motif	P value	Adjusted P value
SRSF2	AGAAG	4.63E-09	4.03E-06
FMR1	GCUGC	6.76E-09	4.03E-06
SFPQ	UGGAGAAC	4.56E-08	1.81E-05
SRSF2	UGUUCCAGAU	2.28E-06	6.79E-04
TRA2A	AAGAAGAA	1.09E-05	1.47E-03
TRA2B	AAGAAGAA	1.09E-05	1.47E-03
NOVA1	UCACC	1.20E-05	1.47E-03
SRSF1	GAAGAAGA	1.36E-05	1.47E-03
SRSF2	GAAGAAGA	1.36E-05	1.47E-03
SRSF4	GAAGAAGA	1.36E-05	1.47E-03
SRSF5	GAAGAAGA	1.36E-05	1.47E-03
SRSF6	GAAGAAGA	1.36E-05	1.47E-03
HNRNPH1	AAGAA	3.88E-05	3.30E-03
HNRNPH2	AAGAA	3.88E-05	3.30E-03
TRA2A	AAGAA	3.88E-05	3.30E-03
TRA2B	AAGAA	3.88E-05	3.30E-03
ZC3H10	GCAGCGC	5.04E-05	4.01E-03
SRSF1	GGAGGAG	8.01E-05	5.73E-03
PABPN1	AGAAGAC	8.16E-05	5.73E-03
SRSF1	AAAAGAGAAG	9.19E-05	5.77E-03
SRSF2	AAAAGAGAAG	9.19E-05	5.77E-03
TRA2B	GAAAGAAG	1.47E-04	8.37E-03
TRA2B	GAAAGAAG	1.47E-04	8.37E-03
NOVA1	AUCACC	2.34E-04	1.21E-02
NOVA2	AUCACC	2.34E-04	1.21E-02
HNRNPA1	CUGAG	2.43E-04	1.21E-02
YBX1	CACCACCACC	2.80E-04	1.26E-02
SRSF3	CACCACCACC	2.80E-04	1.26E-02
KHSRP	CACCCUCC	2.85E-04	1.26E-02
TRA2B	AAGAAGAAG	5.62E-04	2.31E-02
TRA2B	AAGAAGAAG	5.62E-04	2.31E-02
SNRPA	AUUGCAC	6.54E-04	2.60E-02
RBM8A	GCGCGCG	6.76E-04	2.60E-02
SRSF2	UGUUCCAGAU	8.66E-04	3.23E-02
SRSF2	AGCAG	9.03E-04	3.26E-02
IGHMBP2	AAAAAAAA	1.00E-03	3.42E-02

PABPC1	AAAAAAAA	1.00E-03	3.42E-02
SF1	UGCUGCC	1.39E-03	4.55E-02
SRSF1	GAGGAGGA	1.45E-03	4.55E-02
SRSF2	GAGGAGGA	1.45E-03	4.55E-02
SRSF9	AGGAGCA	1.61E-03	4.92E-02
SRSF10	AGAGAAA	1.68E-03	4.96E-02
SRSF11	AAGAAG	1.79E-03	4.96E-02
TRA2B	AAGAAG	1.79E-03	4.96E-02
TRA2B	AAGAAG	1.79E-03	4.96E-02

Supplementary Table 7 GO term enrichment analysis of the RBPs with binding motifs enriched in the precited SVRs (FDR < 0.05). Enrichment analysis was conducted with the background of all the 159 RBPs utilizing the DAVID bioinformatics resources 6.8. P values were calculated using one-sided Fisher's exact test and adjusted with the Benjamini-Hochberg method.

Term	Fold Enrichment	P value	Adjusted P value
mRNA splicing, via spliceosome	2.34	1.14E-07	1.51E-05
RNA export from nucleus	4.26	2.15E-05	1.43E-03
mRNA 3'-end processing	3.97	4.84E-05	1.61E-03
termination of RNA polymerase II transcription	3.97	4.84E-05	1.61E-03
mRNA export from nucleus	3.73	9.94E-05	2.64E-03

Supplementary Table 8 Sensitivity analysis of DiffScan in terms of tuning parameters. For each scenario in the simulations, the coefficient of variation for nine values of mean precision at recall values below 0.05, corresponding to the nine settings of (r, γ): $r \in \{1, 2, 3\}$ and $\gamma \in \{0.4, 0.5, 0.6\}$, is displayed.

Coefficient of variation		Level of strength of differential signals in SVRs		
		Low	Medium	High
Type of reactivity distributions to simulate reactivities	Cordero et al.	0.133	0.059	0.059
	icSHAPE	0.147	0.068	0.086
	Sükösd et al.	0.148	0.082	0.083

Supplementary Figures

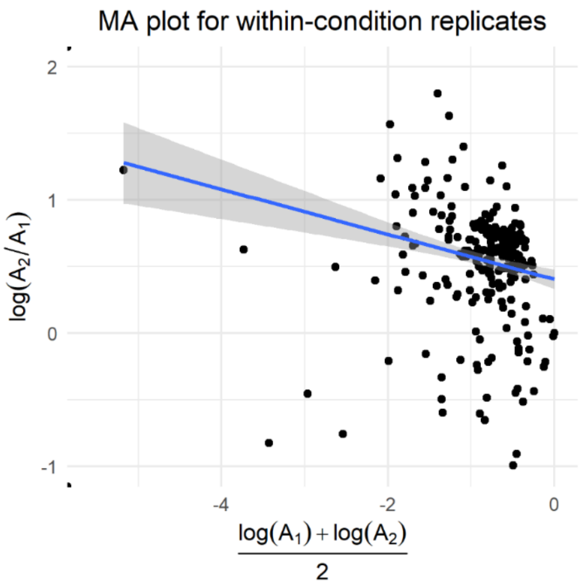

Supplementary Figure 1 Systematic bias between reactivity replicates. $\mathrm{A}_1, \mathrm{A}_2$ are within-condition replicates of the SRP *vivo* dataset (*i.e.*, Control 6; see Materials and Methods). The fitted line should approximate $\log\left(\frac{\mathrm{A}_2}{\mathrm{A}_1}\right) = 0$ if $\mathrm{A}_1, \mathrm{A}_2$ are comparable. The grey band indicates the 95% confidence interval.

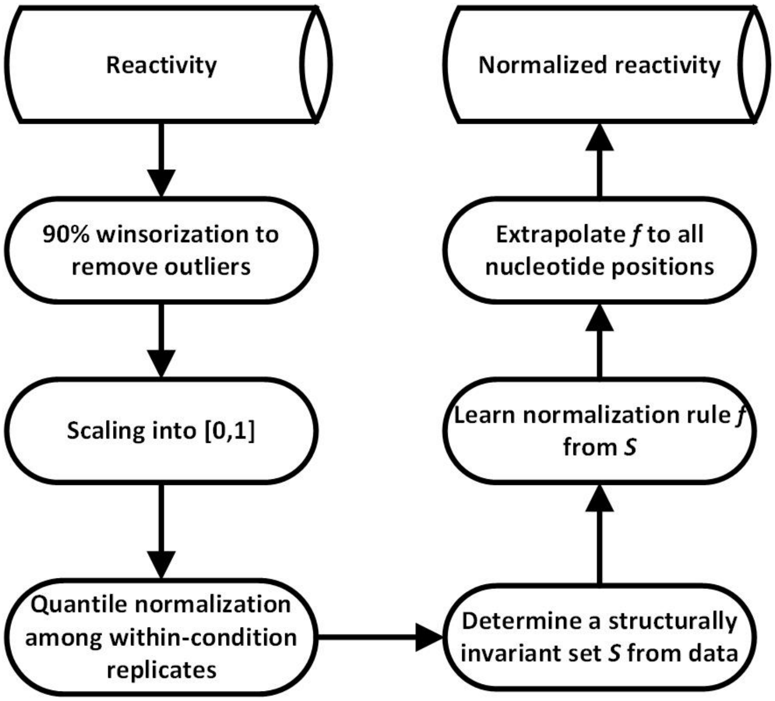

Supplementary Figure 2 Flowchart of the Normalization module.

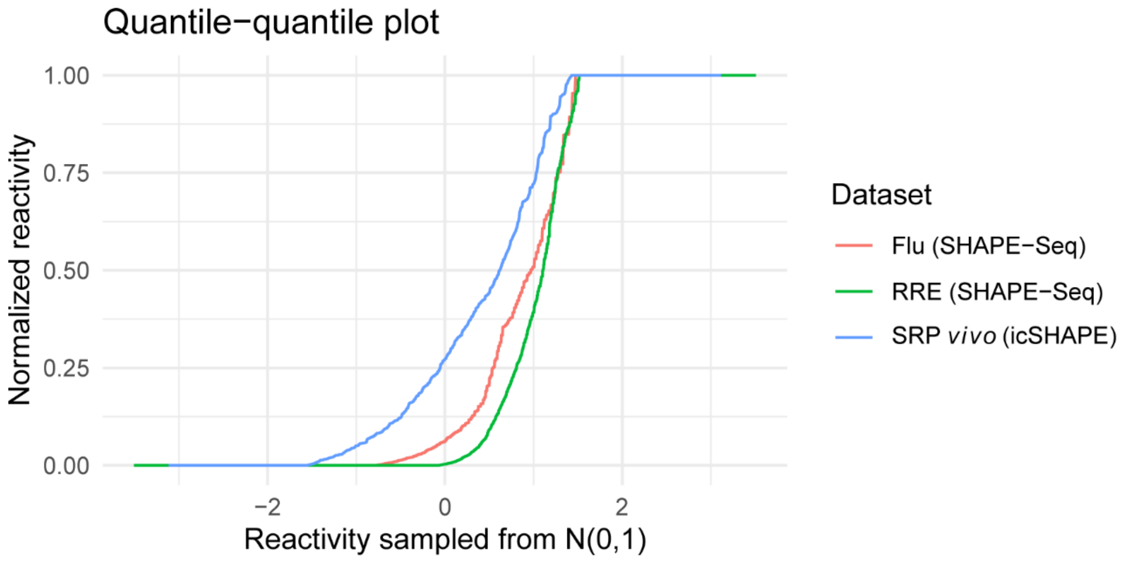

Supplementary Figure 3 Quantile-quantile plot for the normalized reactivities by the Normalization module of DiffScan and reactivities sampled from N(0,1). Normalized reactivities for three real datasets are displayed: the Flu and RRE dataset acquired using the SHAPE-Seq platform and the SRP *vivo* (*i.e.*, Control 6) dataset acquired using the icSHAPE platform (see Materials and Methods). Discrepancies of the distributions of the normalized reactivities from normal distributions, and the differences among the distributions of the normalized reactivities demonstrate the necessity to take consideration of the diverse reactivity distributions from different SP platforms for subsequent differential analysis.

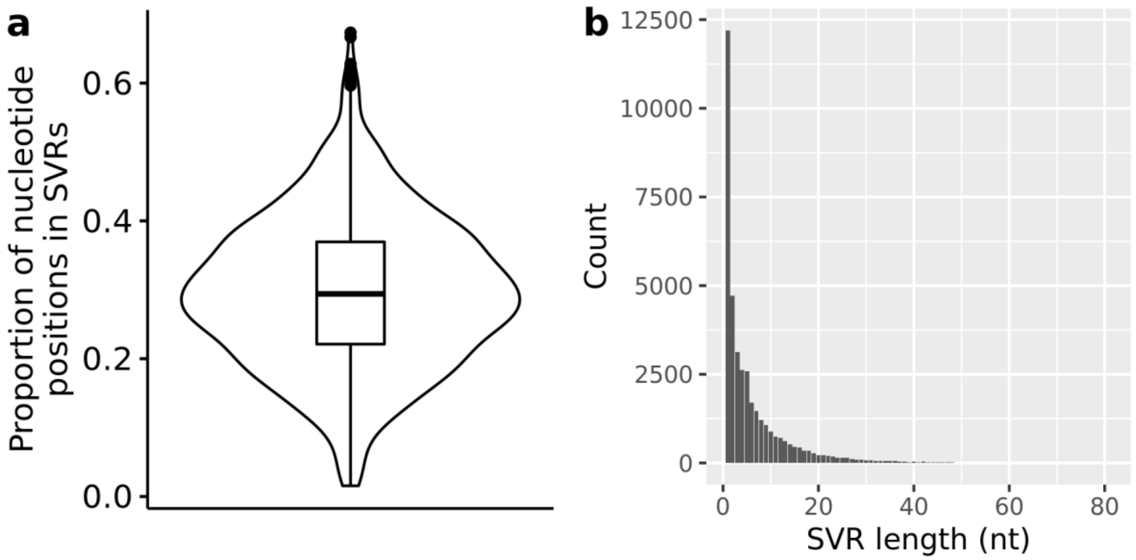

Supplementary Figure 4 Details of the large-scale simulated datasets. a Proportion of nucleotide positions that are in SVRs for each of the 1,000 transcripts. Boxplot elements: center line, median; box limits, upper and lower quartiles; whiskers, 1.5x interquartile range; points, outliers. **b** Length distribution of the simulated SVRs.

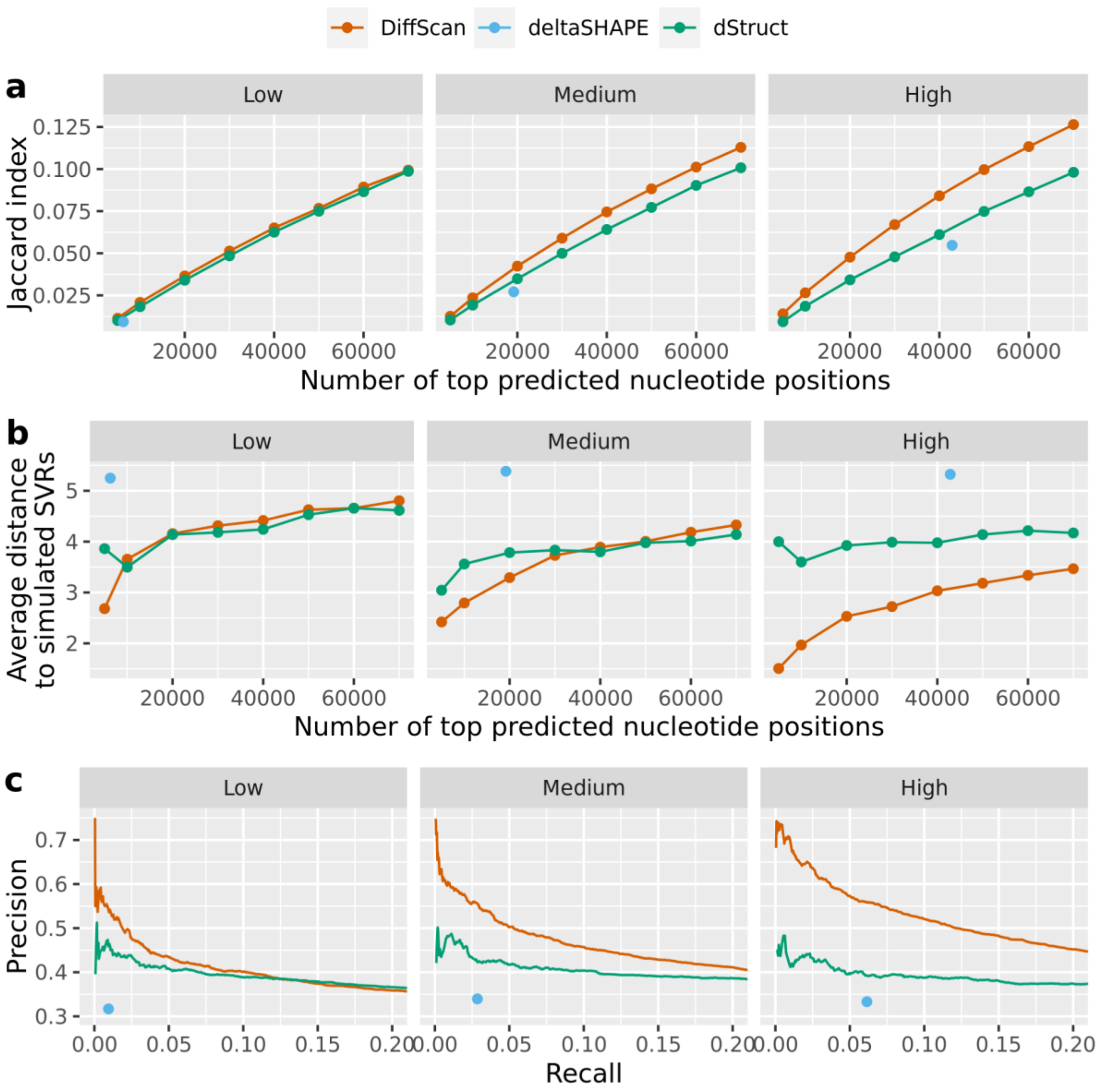

Supplementary Figure 5 Comparison of DiffScan and existing SVR detection methods in simulated datasets (dStruct with minimum search length 1 nt). Default search length of 5 nt is used for deltaSHAPE. The empirical model in Sükösd *et al.*[14] was used to simulate reactivities. **a** Jaccard index between the top predicted nucleotides and the true SVRs at varying cutoffs. **b** Average distance between the top predicted nucleotides and the true SVRs at varying cutoffs. **c** Precision-Recall curves. Columns: three levels of strength of differential signals at simulated SVRs. Note deltaSHAPE does not allow external thresholding, and therefore it is represented as dots instead of curves.

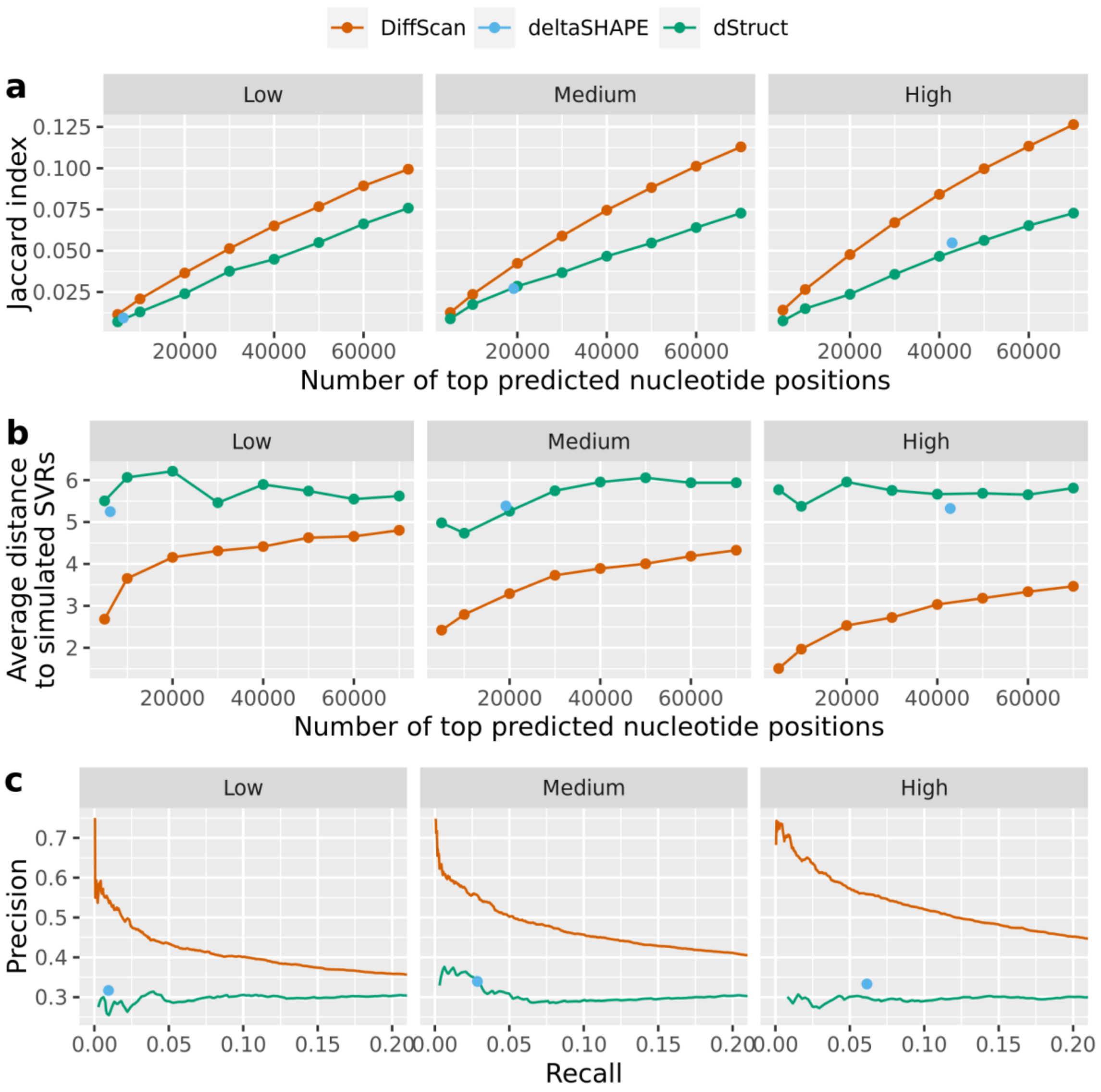

Supplementary Figure 6 Comparison of DiffScan and existing SVR detection methods in simulated datasets (dStruct with minimum search length 11 nt). Default search length of 5 nt is used for deltaSHAPE. The empirical model in Sükösd *et al.*[14] was used to simulate reactivities. **a** Jaccard index between the top predicted nucleotides and the true SVRs at varying cutoffs. **b** Average distance between the top predicted nucleotides and the true SVRs at varying cutoffs. **c** Precision-Recall curves. Columns: three levels of strength of differential signals at simulated SVRs. Note deltaSHAPE does not allow external thresholding, and therefore it is represented as dots instead of curves.

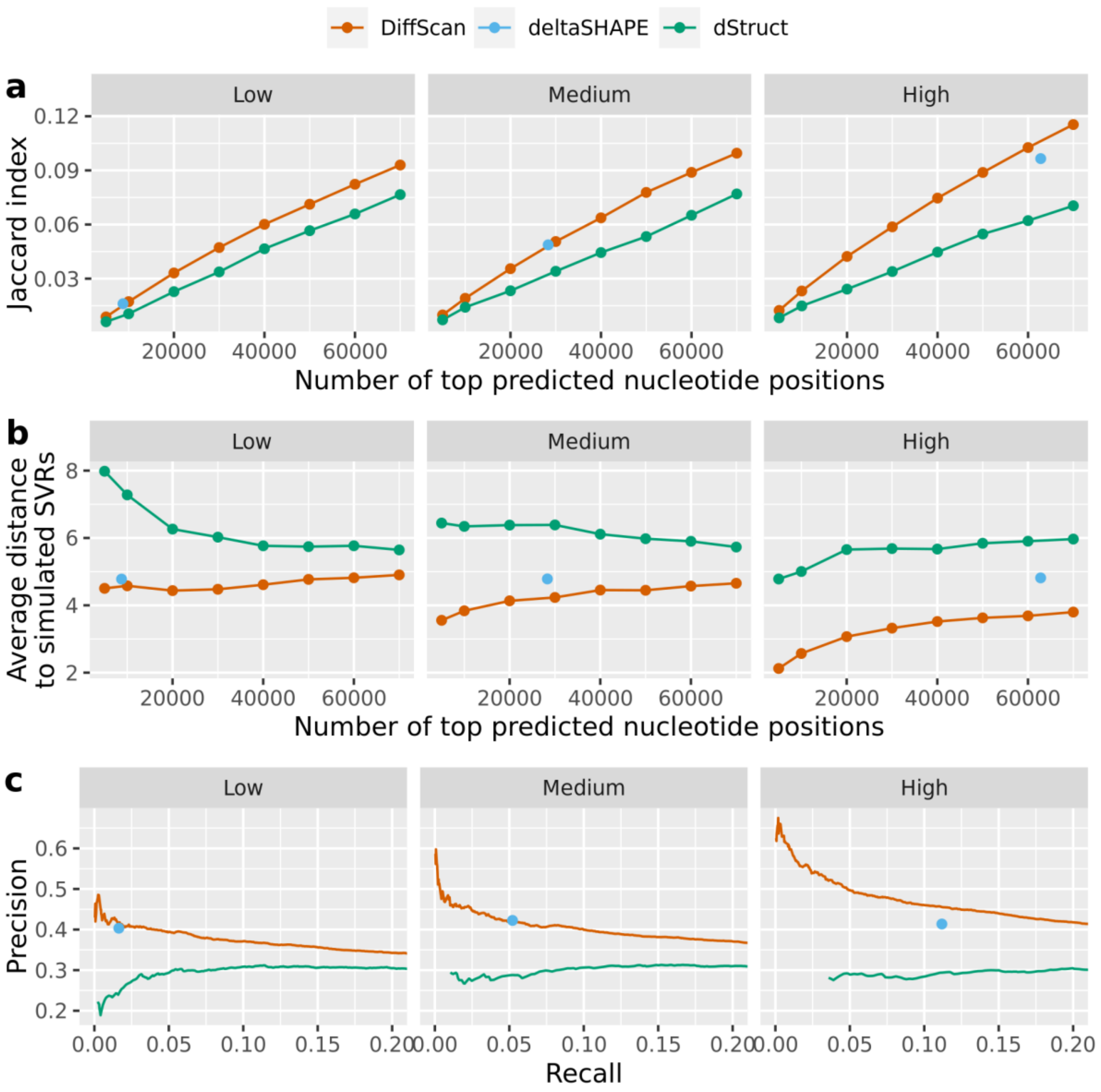

Supplementary Figure 7 Comparison of DiffScan and existing SVR detection methods with the simulated datasets from the empirical model by Cordero *et al*. Default search length of 5 nt is used for deltaSHAPE and minimum search length of 5 nt is used for dStruct. **a** Jaccard index between the top predicted nucleotides and the true SVRs at varying cutoffs. **b** Average distance between the top predicted nucleotides and the true SVRs at varying cutoffs. **c** Precision-Recall curves. Columns: three levels of strength of differential signals at simulated SVRs. Note deltaSHAPE does not allow external thresholding, and therefore it is represented as dots instead of curves.

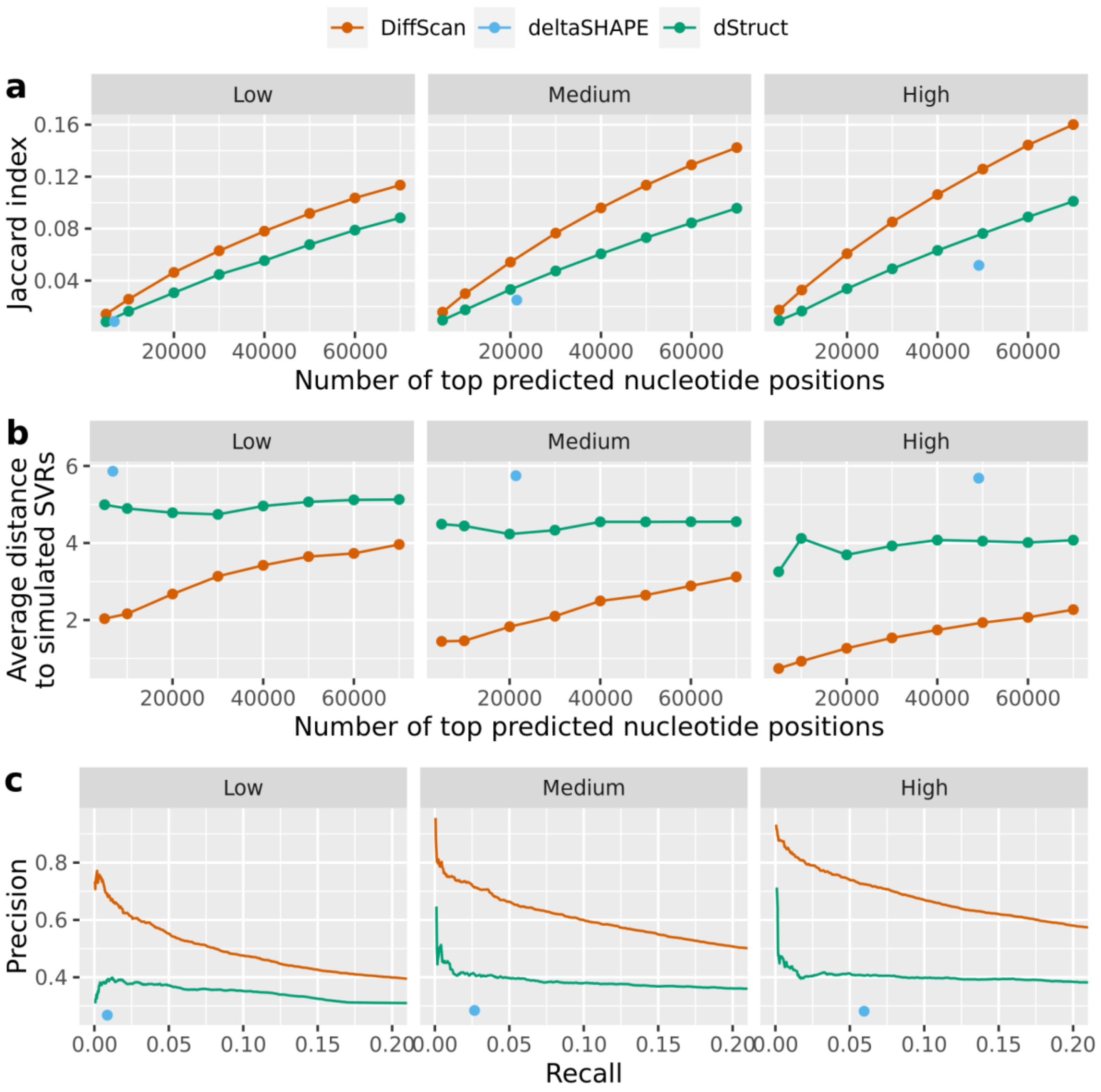

Supplementary Figure 8 Comparison of DiffScan and existing SVR detection methods with the simulated datasets from the empirical model of the icSHAPE platform. Default search length of 5 nt is used for deltaSHAPE and minimum search length of 5 nt is used for dStruct. **a** Jaccard index between the top predicted nucleotides and the true SVRs at varying cutoffs. **b** Average distance between the top predicted nucleotides and the true SVRs at varying cutoffs. **c** Precision-Recall curves. Columns: three levels of strength of differential signals at simulated SVRs. Note deltaSHAPE does not allow external thresholding, and therefore it is represented as dots instead of curves.

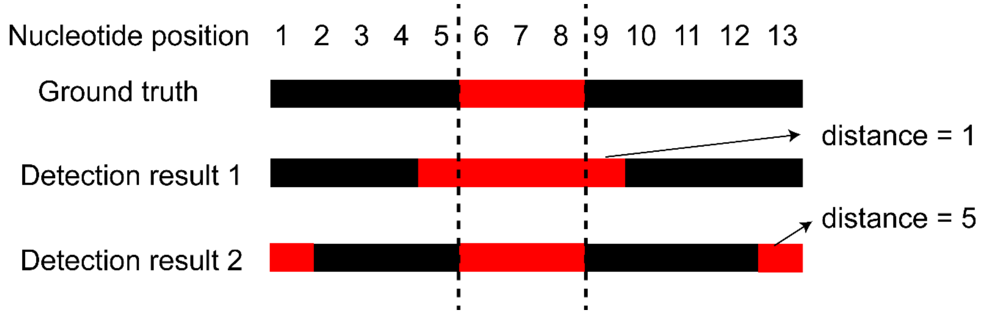

Supplementary Figure 9 An illustration of the definition of the average distance from predicted SVRs and true SVRs. The true SVR covers nucleotide positions 6 nt – 8 nt in the transcript, and Detection result 1 reports a region covering nucleotide position 5 nt – 9 nt. Then the nucleotide distances for Detection result 1 are 1, 0, 0, 0, 1, and the average distance is $\frac{1+0+0+0+1}{5} = 0.4$. In the same way, the average distance for Detection result 2 is $\frac{5+0+0+0+5}{5} = 2$.

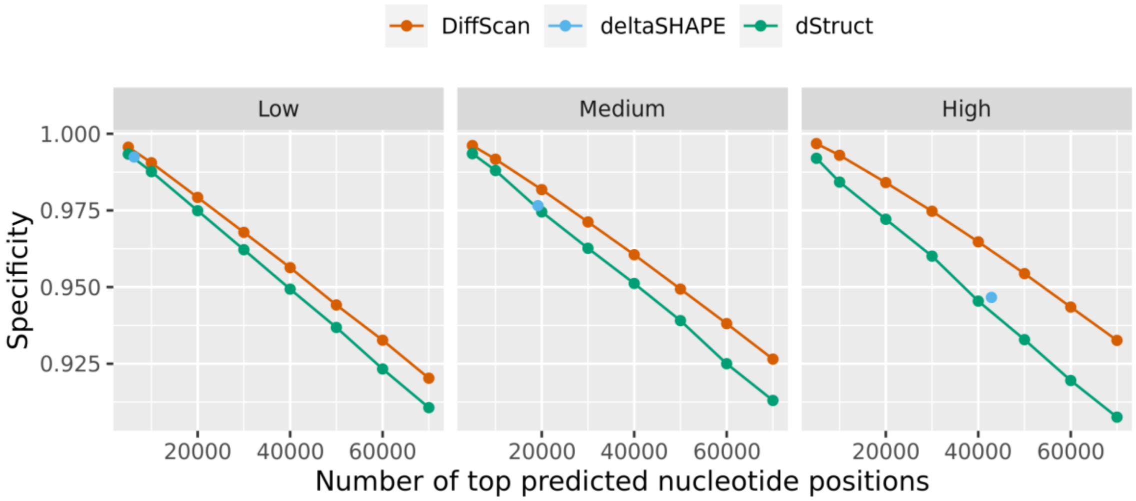

Supplementary Figure 10 Specificity of DiffScan and existing SVR detection methods in simulated datasets. Default search length of 5 nt is used for deltaSHAPE and minimum search length of 5 nt is used for dStruct. The empirical model in Sükösd *et al.*[14] was used to simulate reactivities. Note deltaSHAPE does not allow external thresholding, and therefore it is represented as dots instead of curves.

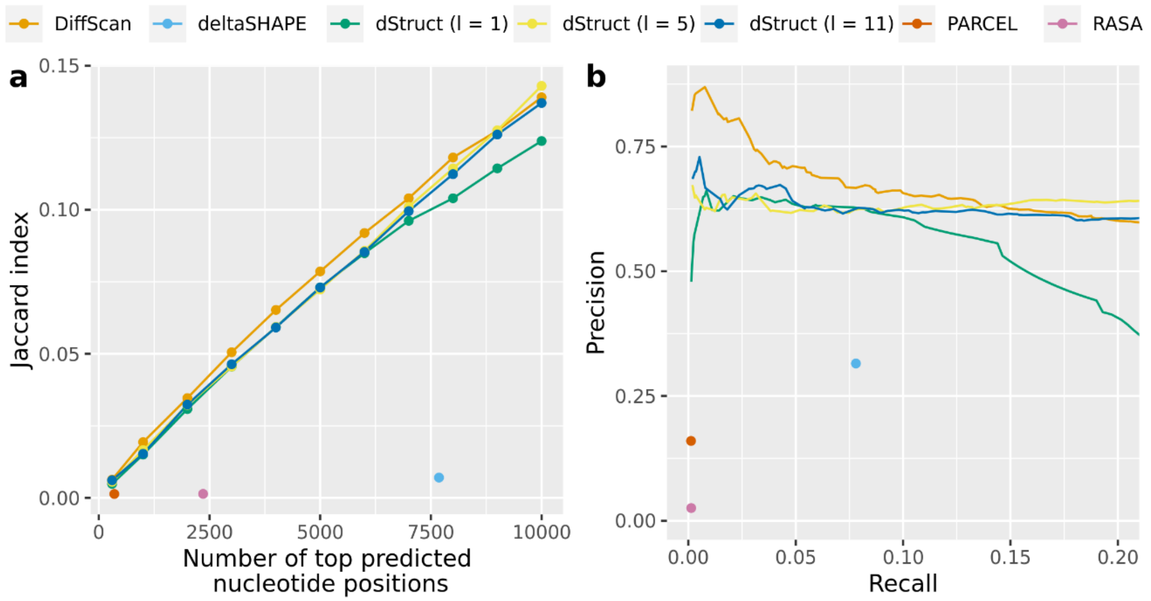

Supplementary Figure 11 Comparison of DiffScan and other SVR detection methods with the simulated dataset by the dStruct paper of Choudhary *et al*. Default search length of 5 nt is used for deltaSHAPE and minimum search length of 1 nt, 5 nt, and 11 nt are used for dStruct. **a** Jaccard index between the top predicted nucleotides and the true SVRs at varying cutoffs. **b** Precision-Recall curves. Note that deltaSHAPE, PARCEL, and RASA do not allow external thresholding, and therefore they are represented as dots instead of curves. diffBUM-HMM is not applicable to the dataset since it requires multiple within-condition replicates of raw count data.

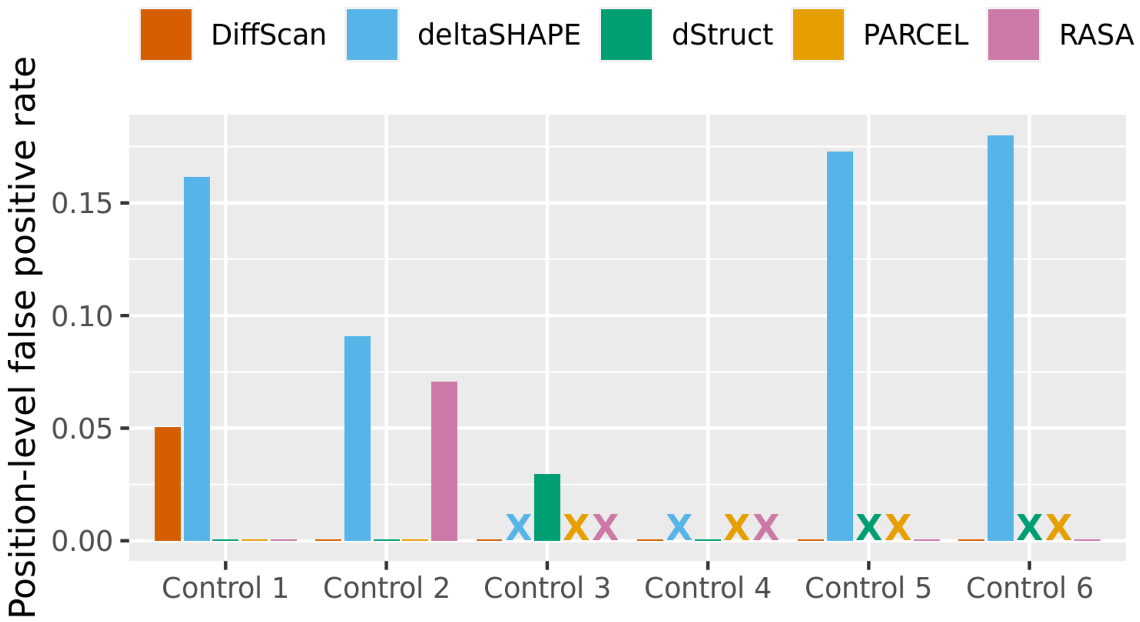

Supplementary Figure 12 Position-level false positive rate at a significance level of 0.05 in six negative control datasets (i.e., datasets having no SVRs). For deltaSHAPE, we used its default search length of 5 nt; for dStruct**,** we used search length of 5 nt following the original article of the method. "X" indicates that the corresponding method was not applicable for the dataset.

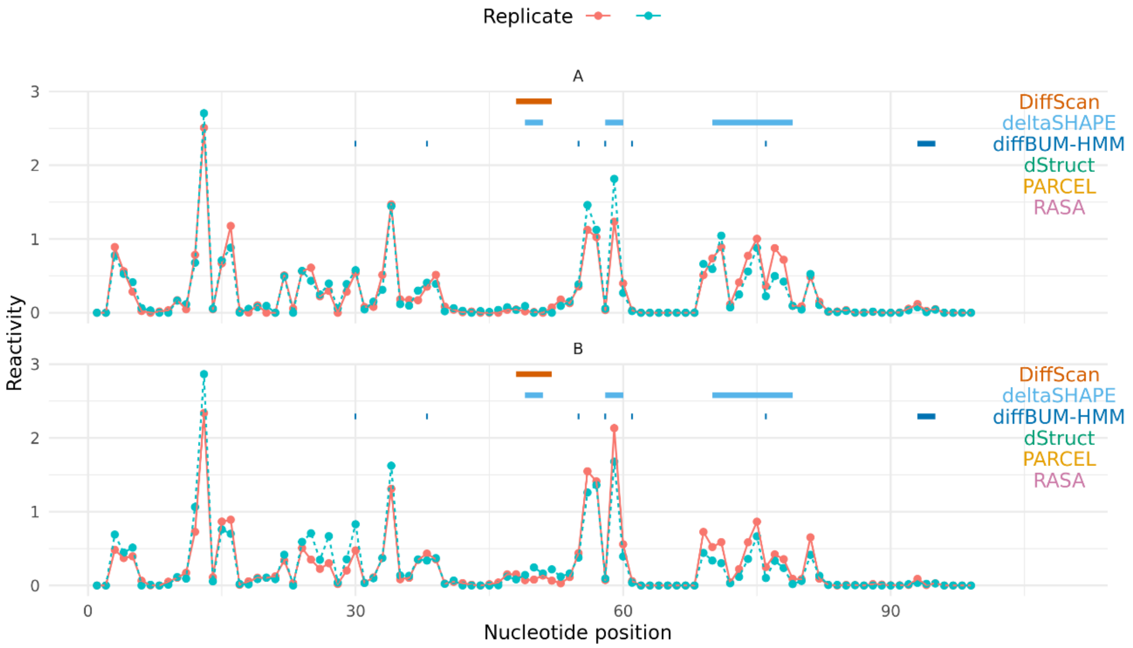

Supplementary Figure 13 The predicted SVRs by different methods for dataset Control 1 which has no SVRs. Top Panel: 2 reactivity replicates in condition A; bottom panel: 2 reactivity replicates in condition B. Line segments at the top denote the predicted SVRs by different methods. DiffScan: family-wide error rate (FWER) < 0.05; diffBUM-HMM: posterior probability > 0.95; dStruct: false discovery rate (FDR) < 0.05.

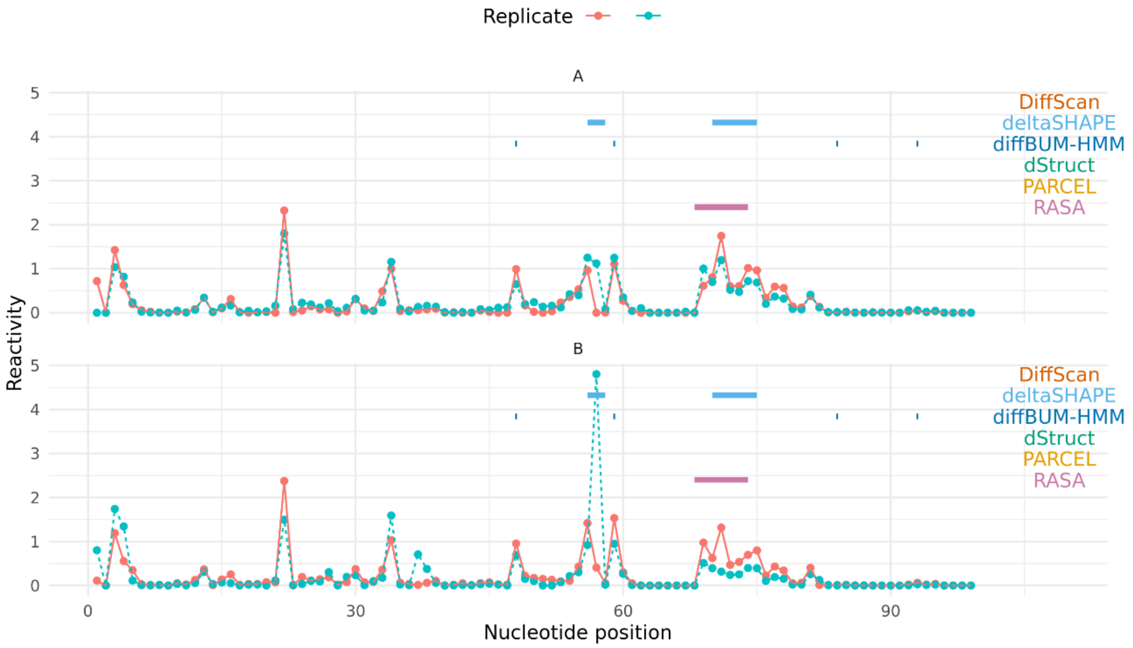

Supplementary Figure 14 The predicted SVRs by different methods for dataset Control 2 which has no SVRs. Top Panel: 2 reactivity replicates in condition A; bottom panel: 2 reactivity replicates in condition B. Line segments at the top denote the predicted SVRs by different methods. DiffScan: family-wide error rate (FWER) < 0.05; diffBUM-HMM: posterior probability > 0.95; dStruct: false discovery rate (FDR) < 0.05.

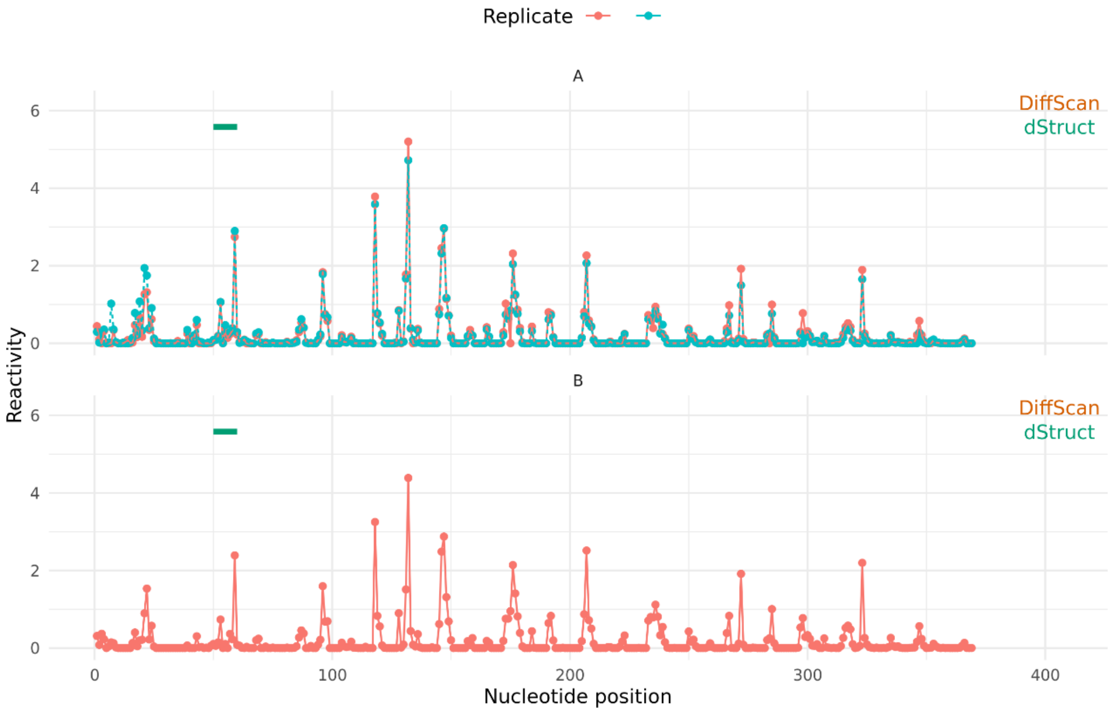

Supplementary Figure 15 The predicted SVRs by different methods for dataset Control 3 which has no SVRs. Top Panel: 2 reactivity replicates in condition A; bottom panel: 1 reactivity replicate in condition B. Line segments at the top denote the predicted SVRs by different methods. DiffScan: family-wide error rate (FWER) < 0.05; dStruct: false discovery rate (FDR) < 0.05. diffBUM-HMM, deltaSHAPE, PACEL, and RASA were not applicable to this dataset.

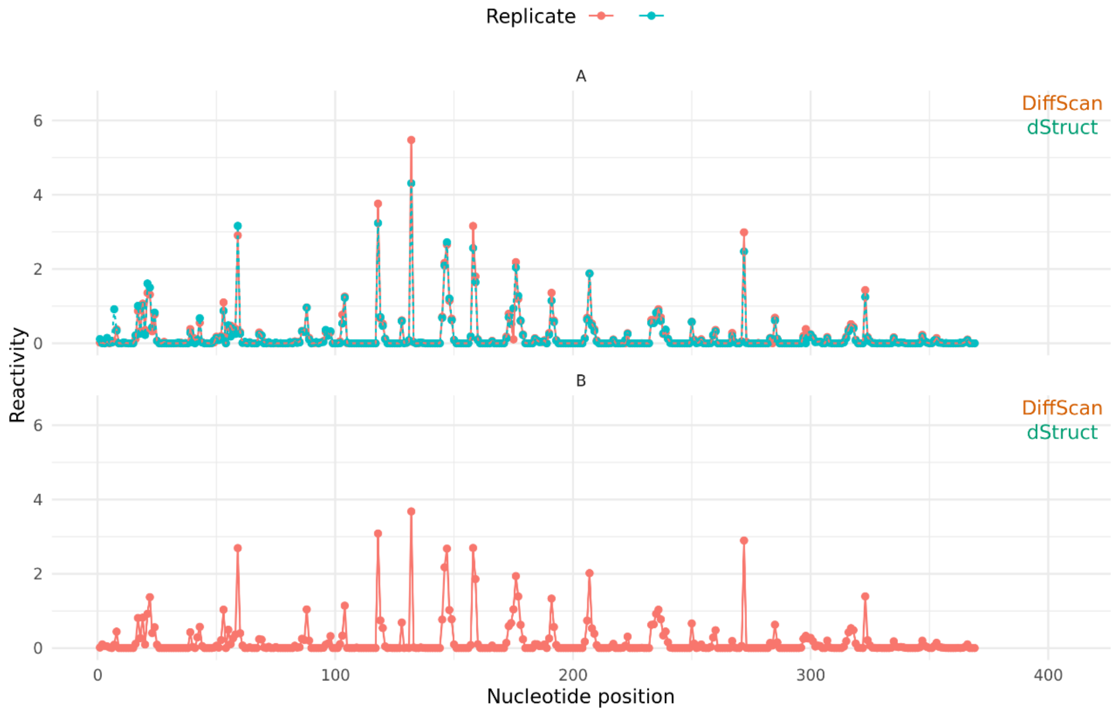

Supplementary Figure 16 The predicted SVRs by different methods for dataset Control 4 which has no SVRs. Top Panel: 2 reactivity replicates in condition A; bottom panel: 1 reactivity replicate in condition B. Line segments at the top denote the predicted SVRs by different methods. DiffScan: family-wide error rate (FWER) < 0.05; dStruct: false discovery rate (FDR) < 0.05. diffBUM-HMM, deltaSHAPE, PACEL, and RASA were not applicable to this dataset.

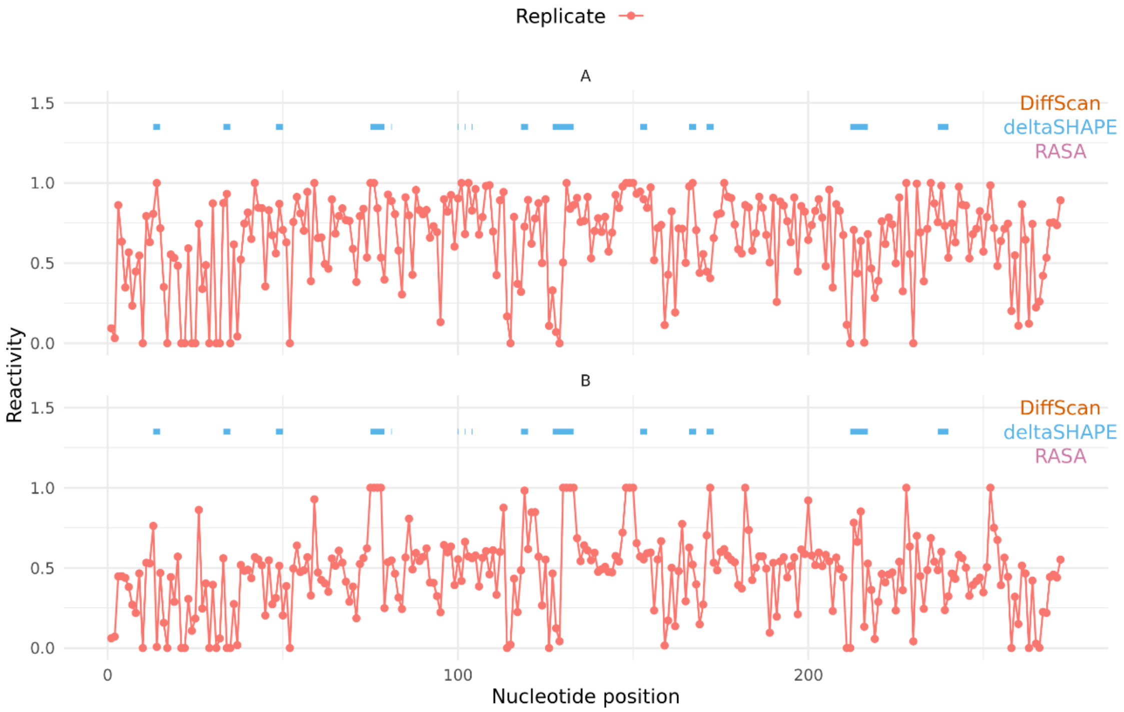

Supplementary Figure 17 The predicted SVRs by different methods for dataset Control 5 which has no SVRs. Top Panel: 1 reactivity replicate in condition A; bottom panel: 1 reactivity replicate in condition B. Line segments at the top denote the predicted SVRs by different methods. DiffScan: family-wide error rate (FWER) < 0.05. diffBUM-HMM, dStruct, and PACEL were not applicable to this dataset due to lack of multiple within-condition replicates.

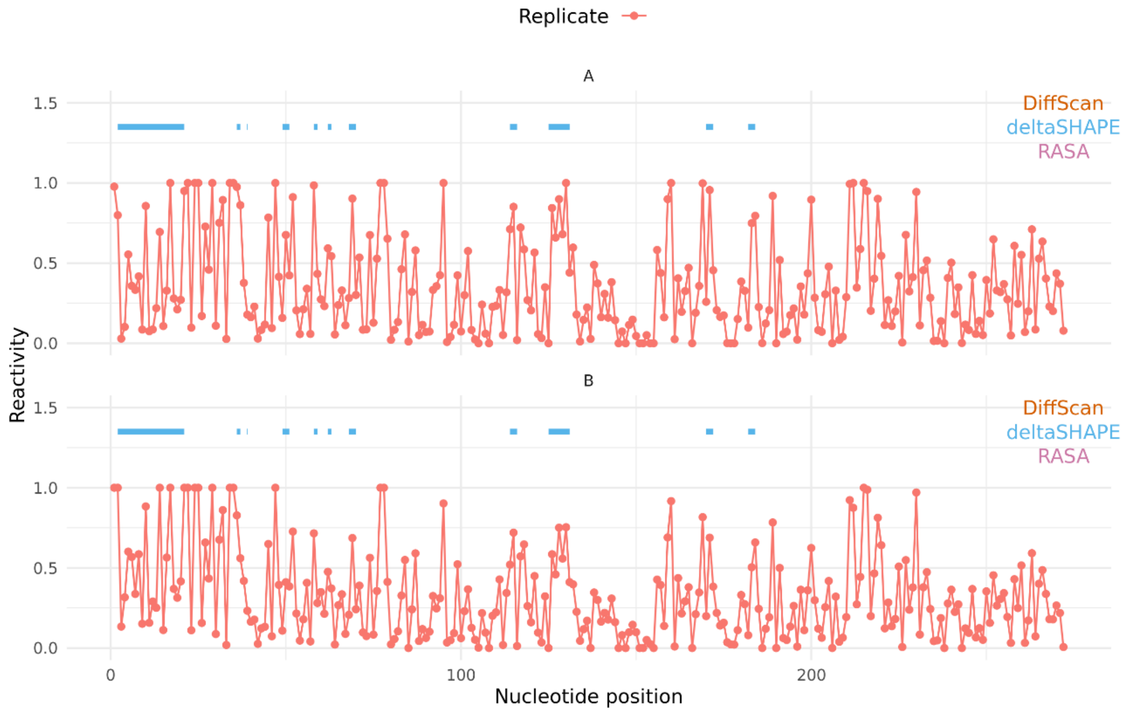

Supplementary Figure 18 The predicted SVRs by different methods for dataset Control 6 which has no SVRs. Top Panel: 1 reactivity replicate in condition A; bottom panel: 1 reactivity replicate in condition B. Line segments at the top denote the predicted SVRs by different methods. DiffScan: family-wide error rate (FWER) < 0.05. diffBUM-HMM, dStruct, and PACEL were not applicable to this dataset due to lack of multiple within-condition replicates.

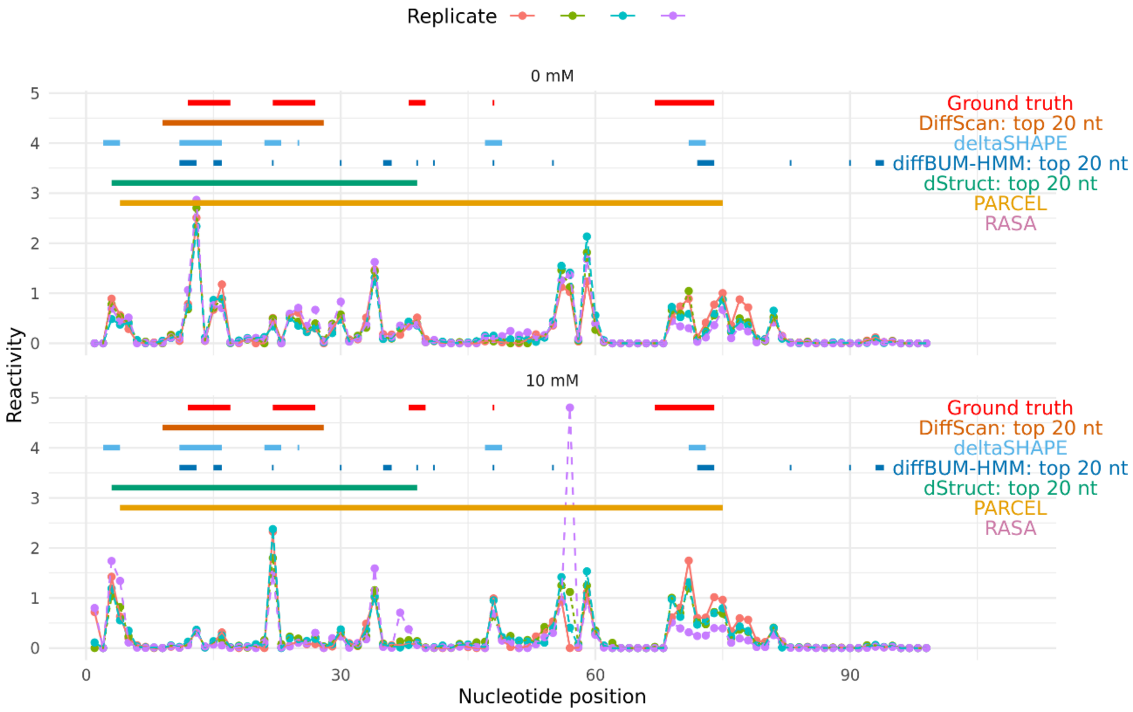

Supplementary Figure 19 Top-20 ranked nucleotide positions by different methods for the Flu dataset. Top Panel: 4 reactivity replicates in the condition of 0 mM fluoride ions; bottom panel: 4 reactivity replicates in the condition of 10 mM fluoride ions. Line segments at the top denote the annotated SVRs and the top-20 ranked nucleotide positions by different methods. (Given a number of top ranked nucleotide positions m, for methods that output predicted SVRs, we sequentially included the top ranked regions until the total number of nucleotide positions in the included regions exceeds or equals to m.) RASA did not report any region.

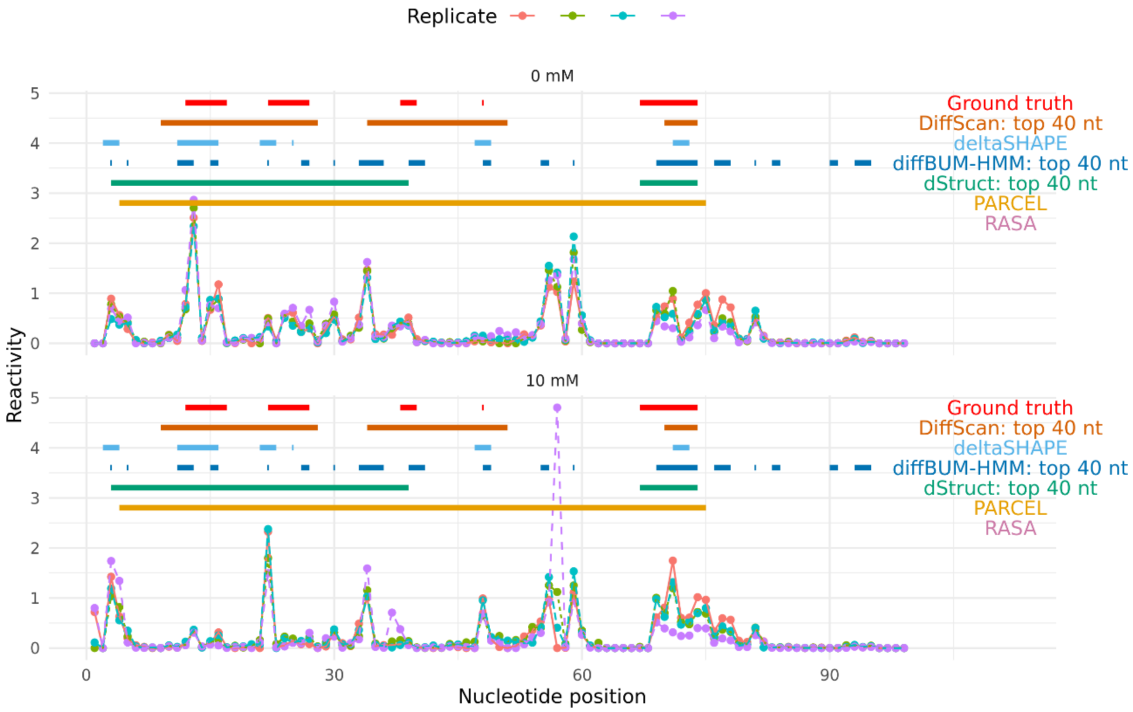

Supplementary Figure 20 Top-40 ranked nucleotide positions by different methods for the Flu dataset. Top Panel: 4 reactivity replicates in the condition of 0 mM fluoride ions; bottom panel: 4 reactivity replicates in the condition of 10 mM fluoride ions. Line segments at the top denote the annotated SVRs and the top-40 ranked nucleotide positions by different methods. (Given a number of top ranked nucleotide positions m, for methods that output predicted SVRs, we sequentially included the top ranked regions until the total number of nucleotide positions in the included regions exceeds or equals to m.) RASA did not report any region.

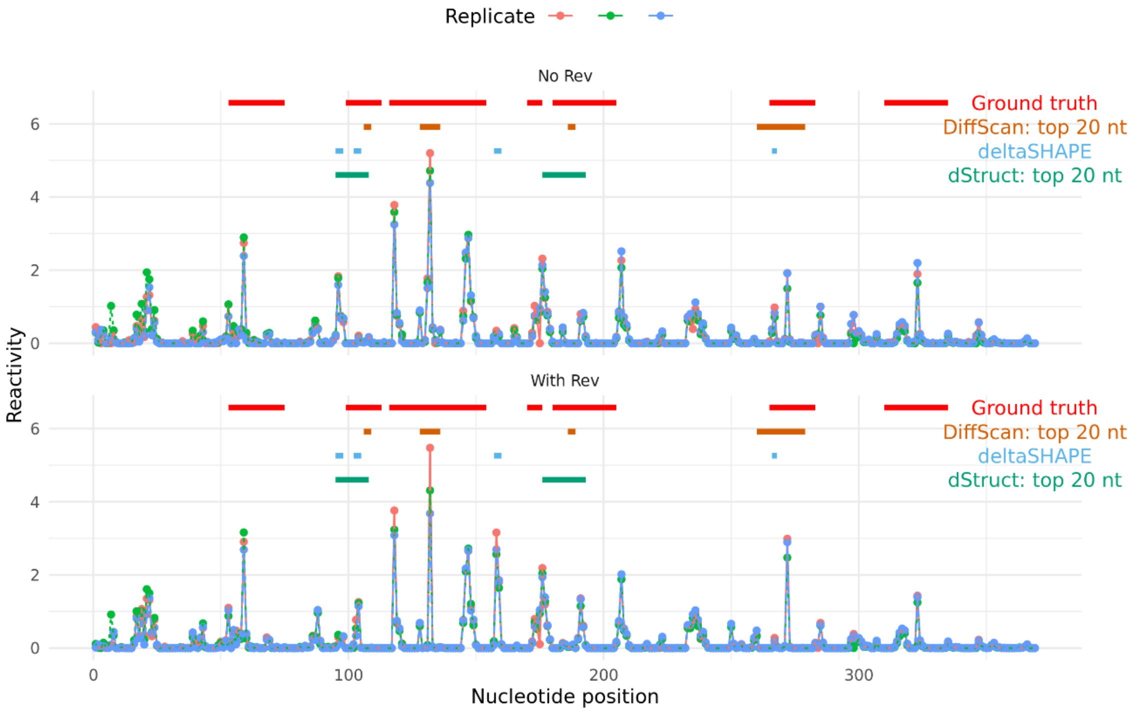

Supplementary Figure 21 Top-20 ranked nucleotide positions by different methods for the RRE dataset. Top Panel: 3 reactivity replicates in the condition with protein Rev; bottom panel: 3 reactivity replicates in the condition without protein Rev. Line segments at the top denote the annotated SVRs and the top-20 ranked nucleotide positions by different methods. (Given a number of top ranked nucleotide positions m, for methods that output predicted SVRs, we sequentially included the top ranked regions until the total number of nucleotide positions in the included regions exceeds or equals to m.) diffBUM-HMM, PARCEL, and RASA was not applicable for the RRE dataset.

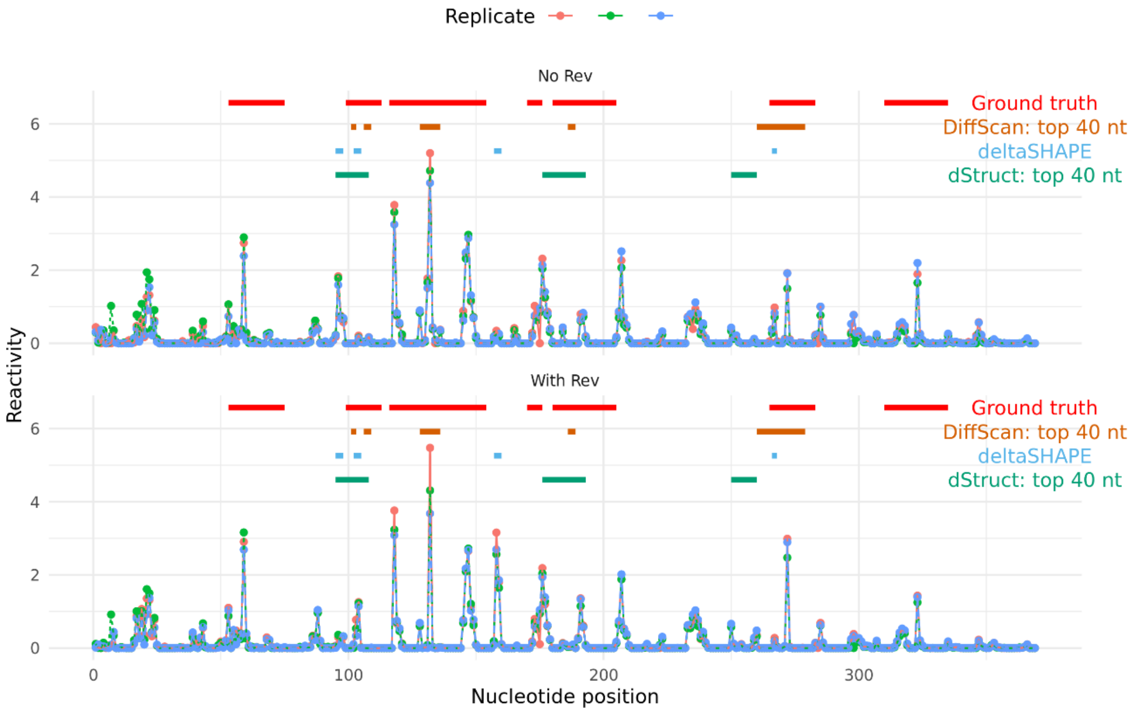

Supplementary Figure 22 Top-40 ranked nucleotide positions by different methods for the RRE dataset. Top Panel: 3 reactivity replicates in the condition with protein Rev; bottom panel: 3 reactivity replicates in the condition without protein Rev. Line segments at the top denote the annotated SVRs and the top-40 ranked nucleotide positions by different methods. (Given a number of top ranked nucleotide positions m, for methods that output predicted SVRs, we sequentially included the top ranked regions until the total number of nucleotide positions in the included regions exceeds or equals to m.) diffBUM-HMM, PARCEL, and RASA was not applicable for the RRE dataset.

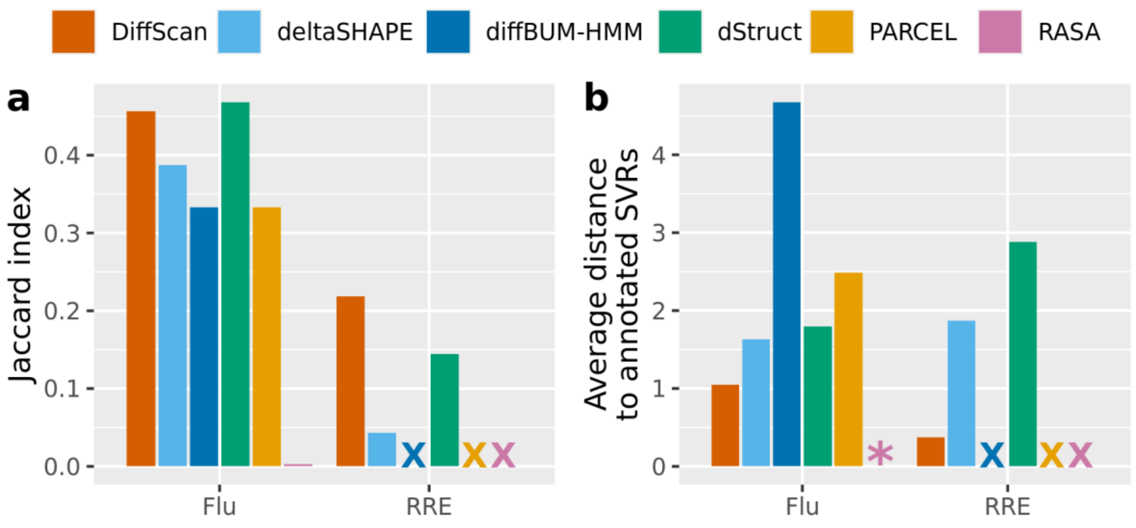

Supplementary Figure 23 Comparison of the top-40 ranked nucleotide positions by different methods in the benchmark datasets. Default search length of 5 nt is used for deltaSHAPE and minimum search length of 5 nt is used for dStruct following the original article of the method. **a** Jaccard index between the top-40 ranked nucleotides and the annotated SVRs. **b** Average distance from the top-40 ranked nucleotides to annotated SVRs. “X” indicates that the corresponding method was not applicable for the dataset. “*” indicates that the average distance cannot be calculated since the corresponding method did not report any region.

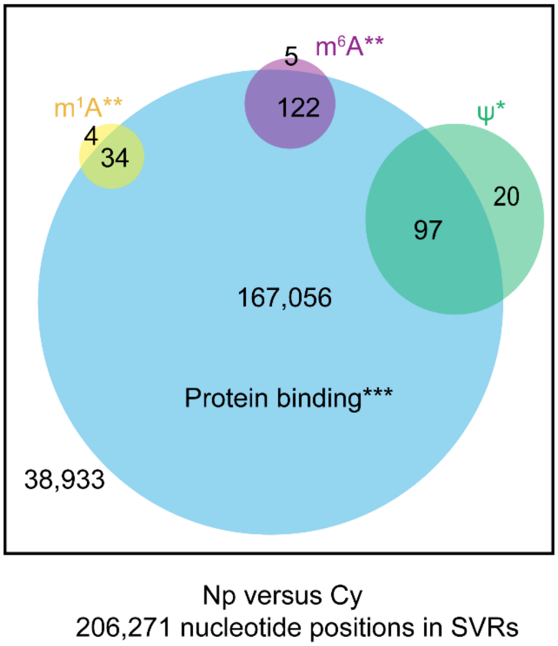

Supplementary Figure 24 Predicted SVRs by DiffScan for Np versus Cy were enriched with protein binding sites and RNA modification sites. Np: nucleoplasm, Cy: cytoplasm. *p value (one-sided Fisher's exact test) < 0.05, **p value < 1e-3, ***p value < 1e-6. P values of enrichment: m^1A = 5.12e-5, m^6A = 1.17e-4, ψ = 4.14e-2, protein binding < 2.2e-16.

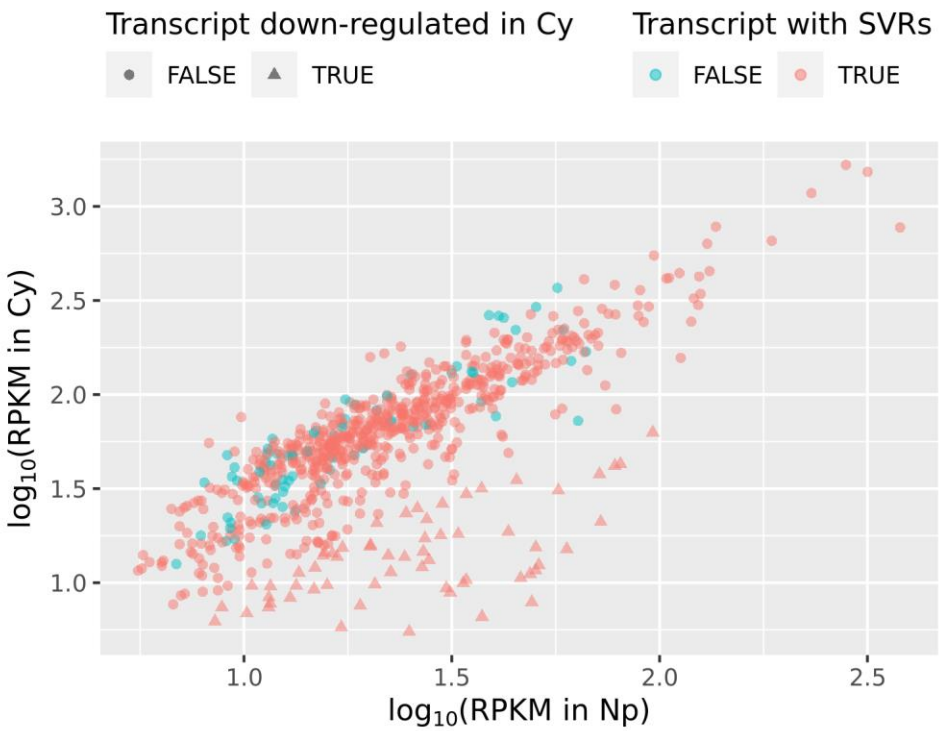

Supplementary Figure 25 RPKM of mRNAs and the prediction results of DiffScan for Np versus Cy. Np: nucleoplasm, Cy: cytoplasm.

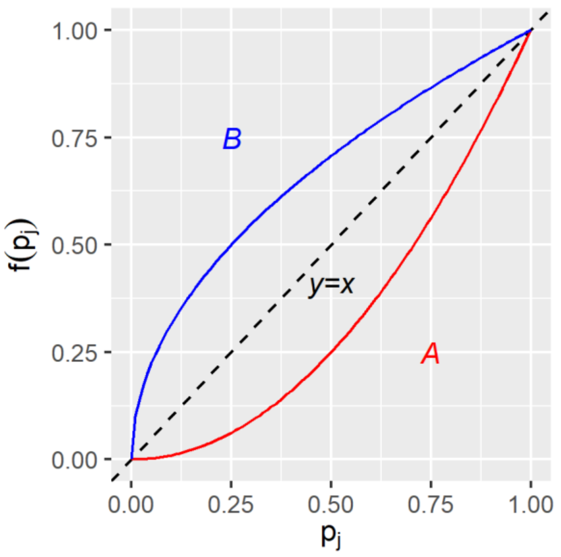

Supplementary Figure 26 An illustrative example for signal to noise ratio in SP data. In experiment A, $\mathrm{f}(\mathrm{p_j}) = \mathrm{p_j^2}$. In experiment B, $\mathrm{f}(\mathrm{p_j}) = \mathrm{p_j}^{1/2}$. Signal to noise ratio in experiment A is higher than that of experiment B.

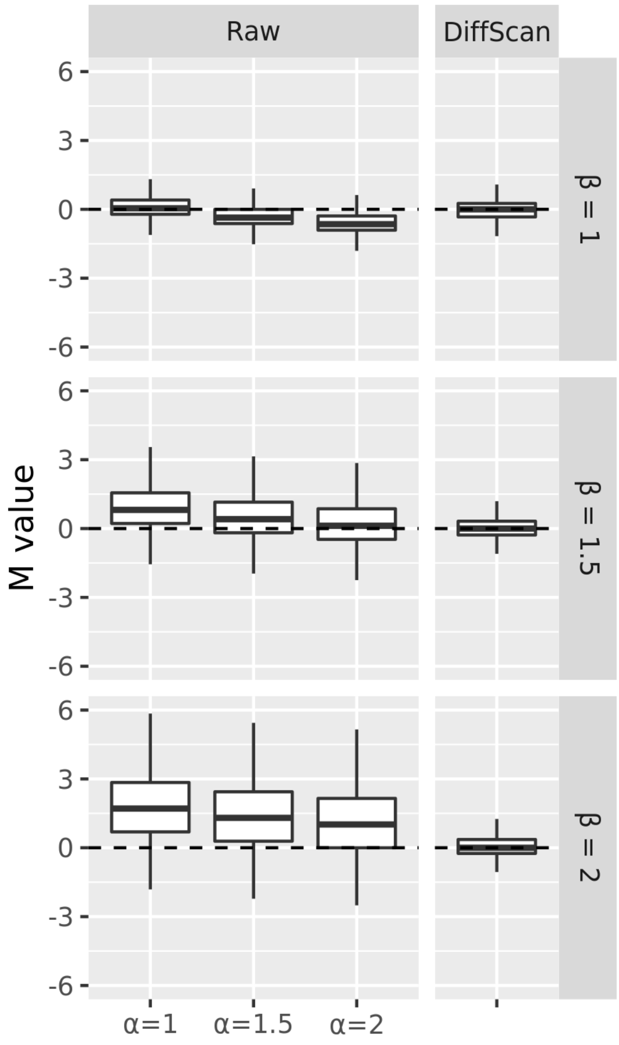

Supplementary Figure 27 Boxplot of M values of raw reactivities and DiffScan-normalized reactivities in the negative control datasets. Boxplot elements: center line, median; box limits, upper and lower quartiles; whiskers, 1.5x interquartile range. n=400 points are plotted for each boxplot, representing the M values at 100 nucleotide positions of the 4 combinations between the 2 replicates of group A and the 2 replicates of group B.

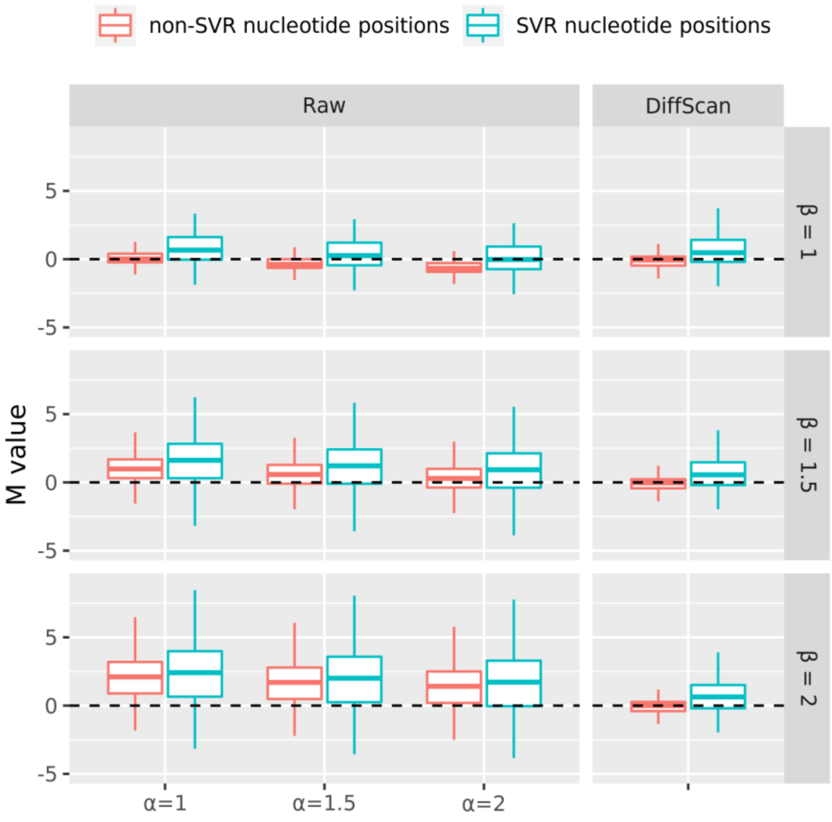

Supplementary Figure 28 Boxplot of M values of raw reactivities and DiffScan-normalized reactivities in the positive control datasets. Boxplot elements: center line, median; box limits, upper and lower quartiles; whiskers, 1.5x interquartile range. n=304 points are plotted for each red boxplot, representing the M values at 76 non-SVR nucleotide positions of the 4 combinations between the 2 replicates of group A and the 2 replicates of group B. n=96 points are plotted for each blue boxplot, representing the M values at 24 SVR nucleotide positions of the 4 combinations between the 2 replicates of group A and the 2 replicates of group B.

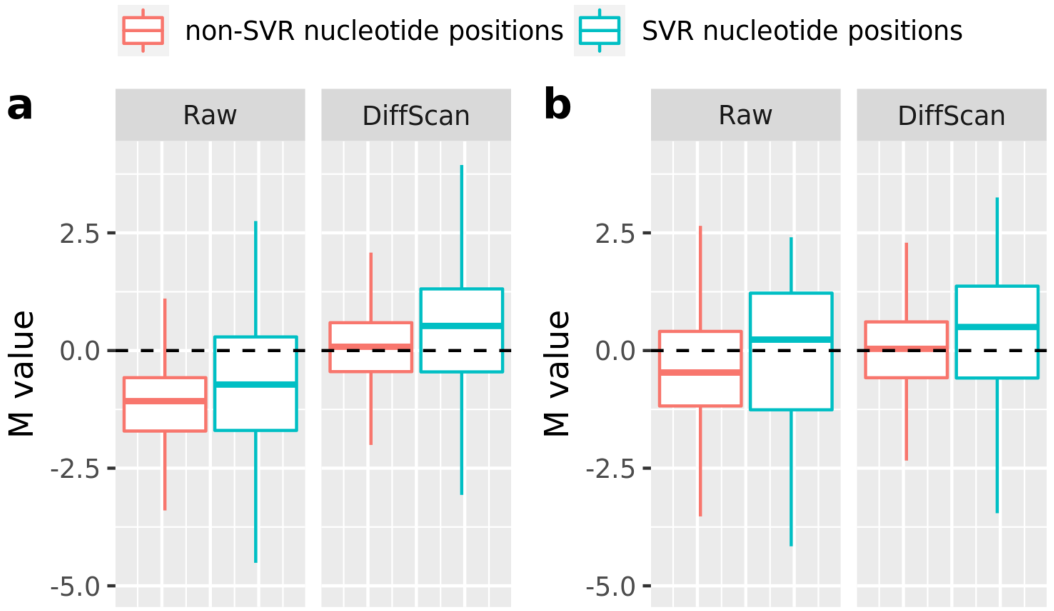

Supplementary Figure 29 Boxplot of M values of raw reactivities and DiffScan-normalized reactivities in benchmark datasets manipulating sequencing depth (a) and signal to noise ratio (b). Boxplot elements: center line, median; box limits, upper and lower quartiles; whiskers, 1.5x interquartile range. n=832 points are plotted for each red boxplot, representing the M values at 52 non-SVR nucleotide positions with known secondary structure of the 16 combinations between the 4 replicates of group A and the 4 replicates of group B. n=384 points are plotted for each blue boxplot, representing the M values at 24 SVR nucleotide positions of the 16 combinations between the 4 replicates of group A and the 4 replicates of group B.

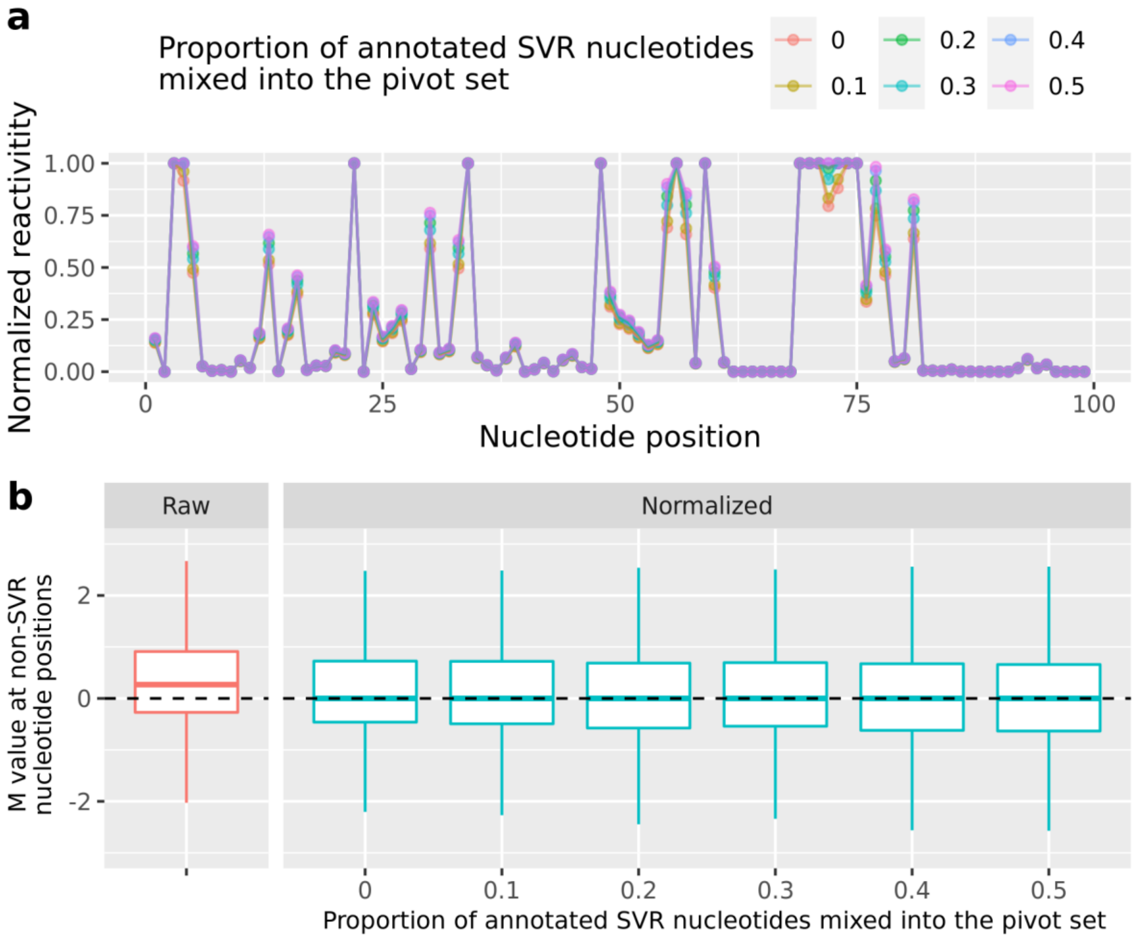

Supplementary Figure 30 Sensitivity analysis of the Normalization module when the pivot set is mis-specified. (**a**) Normalized reactivities by DiffScan when 0%~50% of the nucleotide positions in annotated SVRs were mixed into the true pivot set. (**b**) Boxplot of M values at non-SVR nucleotide positions of raw reactivities and normalized reactivities by DiffScan when 0%~50% of the nucleotide positions in annotated SVRs were mixed into the true pivot set. Boxplot elements: center line, median; box limits, upper and lower quartiles; whiskers, 1.5x interquartile range. n=1,216 points are plotted for each boxplot, representing the M values at 76 non-SVR nucleotide positions of the 16 combinations between the 4 replicates of condition A and the 4 replicates of condition B.

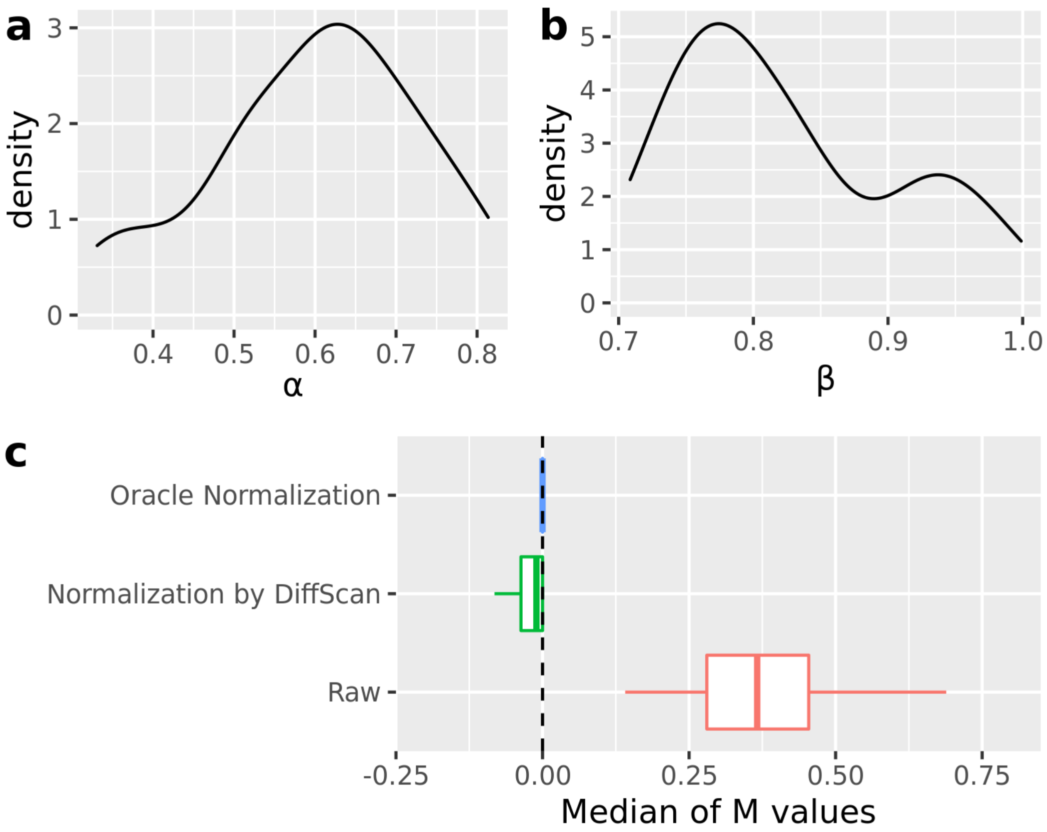

Supplementary Figure 31 Sensitivity analysis of the Normalization module when the difference in sequencing depth and signal to noise ratio between conditions vary along the transcript. (**a-b**) Empirical distributions of α and β fitted from the Flu dataset. (**c**) Boxplot of the median of M values of raw reactivities (red), normalized reactivities by DiffScan (green), and oracle normalization (blue) over 100 replications. Boxplot elements: center line, median; box limits, upper and lower quartiles; whiskers, 1.5x interquartile range. n=400 points are plotted for each boxplot, representing the M values at 100 nucleotide positions of the 4 combinations between the 2 replicates of group A and the 2 replicates of group B.

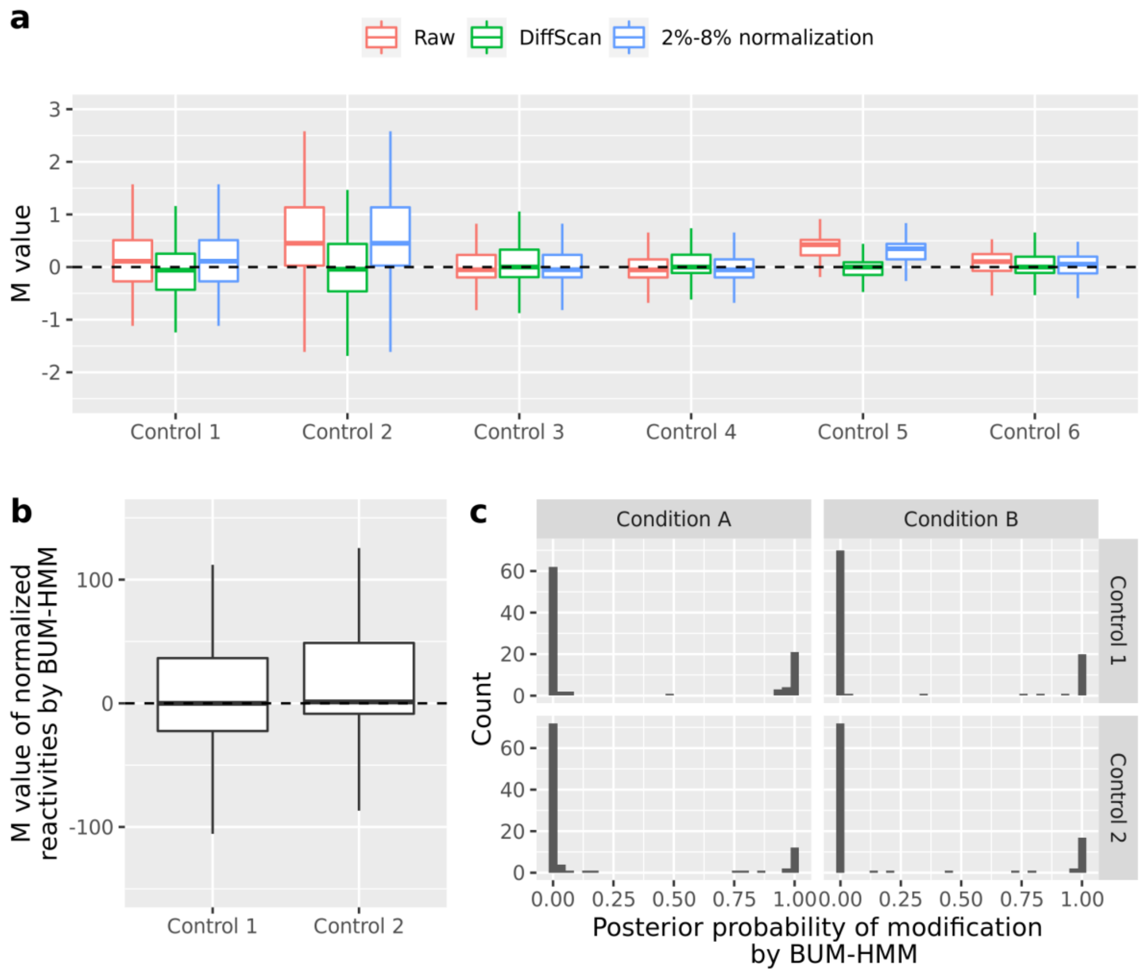

Supplementary Figure 32 Boxplot of M values of raw reactivities and the normalized reactivities by different methods in the datasets Control 1-6. The compared methods are DiffScan, 2%-8% normalization (a), and BUM-HMM (b). The results for BUM-HMM is plotted separately since it is only applicable to Control 1-2, and the range of its M values is substantially different from the other methods. The distribution of the posterior probabilities of BUM-HMM is also provided **(c)**. Boxplot elements: center line, median; box limits, upper and lower quartiles; whiskers, 1.5x interquartile range. Number of points for boxplots in panel (a): n=400 for dataset Control 1 and 2, n=738 for dataset Control 3 and 4, n=272 for dataset Control 5 and 6. Number of points for boxplots in panel (b): n=100 for dataset Control 1 and 2.

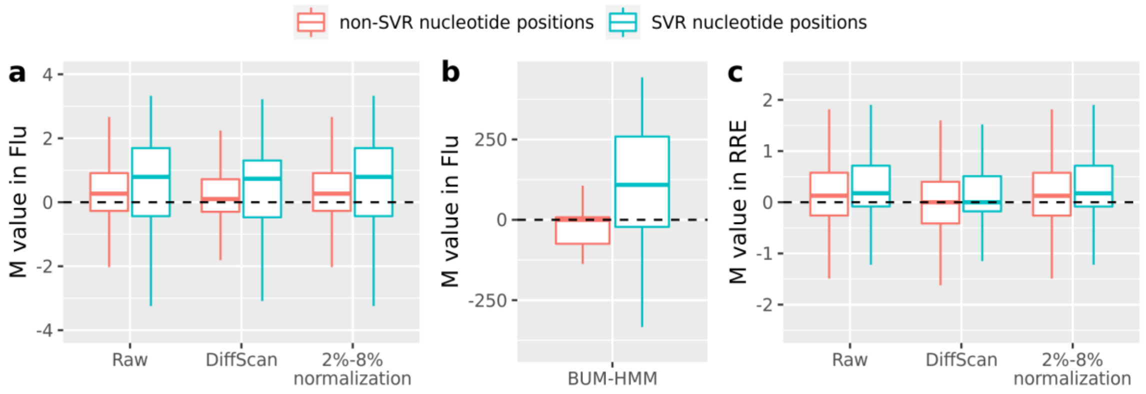

Supplementary Figure 33 Boxplot of M values of the raw and normalized reactivities by different methods in the benchmark datasets Flu (a, b) and RRE (c). BUM-HMM is only applicable to the Flu dataset, and it is separately plotted since the range of its M values is substantially different from the other methods. Boxplot elements: center line, median; box limits, upper and lower quartiles; whiskers, 1.5x interquartile range. Number of points for boxplots in panel (a): n=1,216 for each red boxplot and n=384 for each blue boxplot. Number of points for boxplots in panel (b): n=76 for the red boxplot and n=24 for the blue boxplot. Number of points for boxplots in panel (c): n=1,926 for each red boxplot and n=1,395 for each blue boxplot.

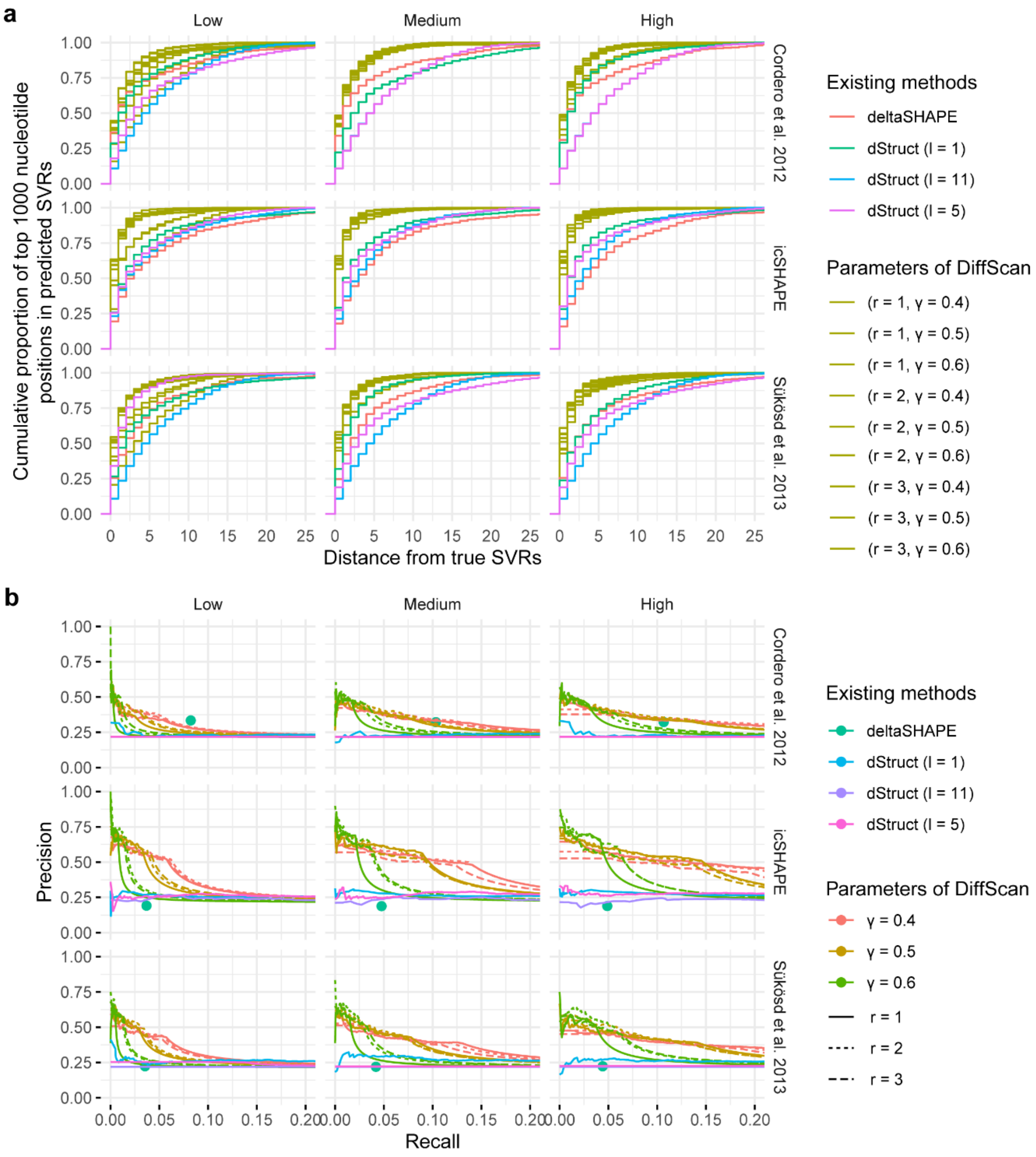

Supplementary Figure 34 DiffScan maintains the relative advantage compared to dStruct and deltaSHAPE in the tested parameter settings. For deltaSHAPE we used the default search length of 5 nt of the method; for dStruct we used search length 1 nt, 5 nt, and 11 nt. **a** Distances between simulated SVRs and top-ranked 1,000 nucleotide positions in predicted SVRs by existing methods and DiffScan with different parameters. **b** Precision-Recall curves for the prediction results from existing methods and DiffScan with different parameters. Rows: three types of reactivity models. Columns: three levels of strength of differential signals at simulated SVRs. Note deltaSHAPE does not allow external thresholding, and therefore it is represented as dots instead of curves.

References

1. Smyth, G.K. in Bioinformatics and Computational Biology Solutions Using R and Bioconductor. (eds. R. Gentleman, V.J. Carey, W. Huber, R.A. Irizarry & S. Dudoit) 397-420 (Springer New York, New York, NY; 2005).
2. Shao, Z., Zhang, Y., Yuan, G.C., Orkin, S.H. & Waxman, D.J. MAnorm: a robust model for quantitative comparison of ChIP-Seq data sets. *Genome Biol* **13**, R16 (2012).
3. Anders, S. & Huber, W. Differential expression analysis for sequence count data. *Genome Biol* **11**, R106 (2010).
4. Augusto, R.d.C. et al. A simple ATAC-seq protocol for population epigenetics. *Wellcome Open Res* **5**, 121-121 (2021).
5. Xiang, G. et al. S3norm: simultaneous normalization of sequencing depth and signal-to-noise ratio in epigenomic data. *Nucleic Acids Res* (2020).
6. Chen, L., Wang, C., Qin, Z.S. & Wu, H. A novel statistical method for quantitative comparison of multiple ChIP-seq datasets. *Bioinformatics* **31**, 1889-1896 (2015).
7. Strobel, E.J., Yu, A.M. & Lucks, J.B. High-throughput determination of RNA structures. *Nat Rev Genet* **19**, 615-634 (2018).
8. Choudhary, K., Lai, Y.H., Tran, E.J. & Aviran, S. dStruct: identifying differentially reactive regions from RNA structurome profiling data. *Genome Biol* **20**, 40 (2019).
9. Low, J.T. & Weeks, K.M. SHAPE-directed RNA secondary structure prediction. *Methods* **52**, 150-158 (2010).
10. Selega, A., Sirocchi, C., Iosub, I., Granneman, S. & Sanguinetti, G. Robust statistical modeling improves sensitivity of high-throughput RNA structure probing experiments. *Nat Methods* **14**, 83-89 (2017).
11. Marangio, P., Law, K.Y.T., Sanguinetti, G. & Granneman, S. diffBUM-HMM: a robust statistical modeling approach for detecting RNA flexibility changes in high-throughput structure probing data. *Genome Biol* **22**, 165 (2021).
12. Kutchko, K.M. & Laederach, A. Transcending the prediction paradigm: novel applications of SHAPE to RNA function and evolution. *Wiley Interdisciplinary Reviews: RNA* **8** (2017).
13. Cordero, P., Kladwang, W., VanLang, C.C. & Das, R. Quantitative dimethyl sulfate mapping for automated RNA secondary structure inference. *Biochemistry* **51**, 7037-7039 (2012).
14. Sukosd, Z., Swenson, M.S., Kjems, J. & Heitsch, C.E. Evaluating the accuracy of SHAPE-directed RNA secondary structure predictions. *Nucleic Acids Res* **41**, 2807-2816 (2013).
15. Sun, L. et al. RNA structure maps across mammalian cellular compartments. *Nat Struct Mol Biol* **26**, 322-330 (2019).
16. Scrucca, L., Fop, M., Murphy, T.B. & Raftery, A.E. mclust 5: Clustering, Classification and Density Estimation Using Gaussian Finite Mixture Models. *R j* **8**, 289-317 (2016).
